# Vaccine-induced systemic and mucosal T cell immunity to SARS-CoV-2 viral variants

**DOI:** 10.1073/pnas.2118312119

**Published:** 2022-05-13

**Authors:** Brock Kingstad-Bakke, Woojong Lee, Shaswath S. Chandrasekar, David J. Gasper, Cristhian Salas-Quinchucua, Thomas Cleven, Jeremy A. Sullivan, Adel Talaat, Jorge E. Osorio, M. Suresh

**Affiliations:** ^a^Department of Pathobiological Sciences, University of Wisconsin–Madison, Madison, WI 53706

**Keywords:** SARS-CoV-2, vaccine, T cells, antibodies, immunity

## Abstract

The first-generation COVID-19 vaccines have been effective in mitigating severe illness and hospitalization, but recurring waves of infections are associated with the emergence of SARS-CoV-2 variants that display progressive abilities to evade antibodies, leading to diminished vaccine effectiveness. The lack of clarity on the extent to which vaccine-elicited mucosal or systemic memory T cells protect against such antibody-evasive SARS-CoV-2 variants remains a critical knowledge gap in our quest for broadly protective vaccines. Using adjuvanted spike protein–based vaccines that elicit potent T cell responses, we assessed whether systemic or lung-resident CD4 and CD8 T cells protected against SARS-CoV-2 variants in the presence or absence of virus-neutralizing antibodies. We found that 1) mucosal or parenteral immunization led to effective viral control and protected against lung pathology with or without neutralizing antibodies, 2) protection afforded by mucosal memory CD8 T cells was largely redundant in the presence of antibodies that effectively neutralized the challenge virus, and 3) “unhelped” mucosal memory CD8 T cells provided no protection against the homologous SARS-CoV-2 without CD4 T cells and neutralizing antibodies. Significantly, however, in the absence of detectable virus-neutralizing antibodies, systemic or lung-resident memory CD4 and “helped” CD8 T cells provided effective protection against the relatively antibody-resistant B1.351 (β) variant, without lung immunopathology. Thus, induction of systemic and mucosal memory T cells directed against conserved epitopes might be an effective strategy to protect against SARS-CoV-2 variants that evade neutralizing antibodies. Mechanistic insights from this work have significant implications in the development of T cell–targeted immunomodulation or broadly protective SARS-CoV-2 vaccines.

## Significance

Immunity induced by the first-generation COVID-19 vaccines may not provide effective and durable protection, either due to waning immunity or due to poor antibody cross-reactivity to new variants. Typically, T cells recognize conserved nonmutable viral epitopes and development of T cell–based vaccines might provide broad immunity to SARS-CoV-2 variants. In this study, we show that adjuvanted spike protein–based experimental vaccines elicited potent respiratory or systemic CD4 and CD8 T cell memory and protected against SARS-CoV-2, in the absence of virus-neutralizing antibodies. Thus, development of T cell–based vaccines might be key to protect against antibody-escape SARS-CoV-2 variants that can potentially overcome immunity induced by current vaccines.

Severe acute respiratory syndrome coronavirus 2 (SARS-CoV-2) has continued to exert devastating impacts on the human life, with >280 million infections and over 5.4 million deaths to date. Although there are millions of convalescent people with some measure of immunity and 8.8 billion doses of vaccine administered to date, further threats of widespread severe COVID-19 disease looms heavily as immunity induced by infection or the first-generation vaccines may not provide effective and durable protection, either due to waning immunity or due to poor antibody cross-reactivity to new variants (1–5).

It is clear that virus-neutralizing antibodies provide the most effective protection to SARS-CoV-2, following vaccination or recovery from infection (6). However, T cell–based protection against SARS-CoV-2 has become a central focus because T cells recognize short amino acid sequences that can be conserved across viral variants (7–9). Indeed, T cells in convalescent COVID-19 patients have shown robust responses that are directed at multiple viral proteins, and depletion of these T cells delayed SARS-CoV-2 control in mice (10–12). These data suggest a protective role for T cells in COVID-19 infection. In effect, what constitutes an effective, an ineffective, or a perilous T cell response to SARS-CoV-2 in lungs remains poorly defined. Controlled studies in laboratory animals are of critical importance to elucidate the role and nature of T cells in lungs during SARS-CoV-2 virus infection and in protective immunity.

Based on the differentiation state, anatomical localization and traffic patterns, memory T cells are classified into effector memory (T_EM_), central memory (T_CM_), and tissue-resident memory (T_RM_) (13, 14). There is accumulating evidence that airway/lung-resident T_RM_s, and not migratory memory T cells (T_EM_s) are critical for protective immunity to respiratory mucosal infections with viruses, such as influenza A virus (IAV) and respiratory syncytial virus (15–21). Development of T_RM_s from effector T cells in the respiratory tract requires local antigen recognition and exposure to critical factors, such as transforming growth factor (TGF)-β and interleukin (IL)-15 (15). Therefore, mucosal vaccines are more likely to elicit T_RM_s in lungs than parenteral vaccines (22, 23). A subset of effector T cells in airways of COVID-19 patients display T_RM_-like features (24), but the development of T_RM_s or their importance in protective immunity to reinfection are yet to be determined. Furthermore, all SARS-CoV-2 vaccines in use are administered parenterally and less likely to induce lung T_RM_s. While depletion of CD8 T cells compromised protection against COVID-19 in vaccinated rhesus macaques (25), the relative effectiveness of vaccine-induced systemic/migratory CD8 T cell memory vs. lung/airway T_RM_s in protective immunity to COVID-19 is yet to be defined.

In this study, using the K18-hACE2 transgenic (tg) mouse model of SARS-CoV-2 infection, we have interrogated two key aspects of T cell immunity: 1) the requirements for lung-resident vs. migratory T cell memory in vaccine-induced immunity to SARS-CoV-2; and 2) the role of lung-resident memory CD4 vs. CD8 T cells in protection against viral variants in the presence or absence of virus-neutralizing antibodies. Studies of mucosal versus systemic T cell–based vaccine immunity using a subunit protein-based adjuvant system that elicits neutralizing antibodies and T cell immunity, demonstrated that: 1) both mucosal and parenteral vaccinations provide effective immunity to SARS-CoV-2 variants; 2) CD4 T cell–dependent immune mechanisms exert primacy in protection against homologous SARS-CoV-2 strain; and 3) the development of spike (S) protein-specific “unhelped” memory CD8 T cells in the respiratory mucosa are insufficient to protect against a lethal challenge with the homologous Washington (WA) strain of SARS-CoV-2. Unexpectedly, we found that systemic or mucosal lung-resident memory CD4 and “helped” CD8 T cells engendered effective immunity to the South African B1.351 β-variant in the apparent absence of detectable mucosal or circulating virus-neutralizing antibodies. Taken together, mechanistic insights from this study have advanced our understanding of viral pathogenesis and might drive rational development of next-generation broadly protective SARS-CoV-2 vaccines that induce humoral and T cell memory.

## Results

### Identification of an Immune Dominant CD8 T Cell Epitope in Responses to SARS-CoV-2 Virus Infection.

**Fig. 1.**
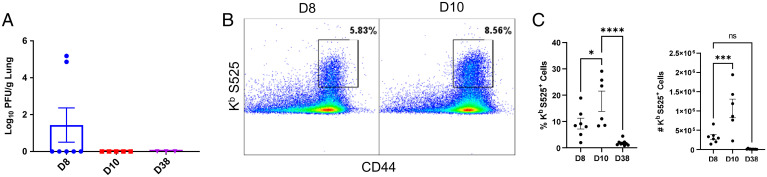
Primary effector and memory T cell responses to SARS-CoV-2 virus in K18-ACE2tg mice. K18-ACE2tg mice were IN infected with 100 PFU of the USA-WA1/2020 (WA strain) of SARS-CoV-2. (*A*) Following SARS-CoV-2 infection, virus titers were measured in lungs. Single-cell suspensions of lungs were stained with viability dye, followed by K^b^/S525 (VNFNFNGL) tetramers in combination with anti-CD4, CD8, and CD44. (*B* and *C*) Frequencies and numbers of S525-specific CD8 T cells in the lungs among gated CD8 T cells are shown in FACS plots and graphs. Data represent four independent experiments. Asterisks indicate significance at *P < 0.05, ***P < 0.0005, and ****P < 0.00005; ns, not significant. Data in each graph indicate mean ± SEM.

We first infected K18-hACE2tg mice with a sublethal dose of the USA-WA1/2020 strain of SARS-CoV-2 (100 PFU) and found that viral control occurred by day 10 postchallenge (Fig. 1*A*). Our in silico analysis revealed that the K^b^-restricted CD8 T cell epitope VNFNFNGL S525-532 (S525) in the SARS-CoV S protein was conserved in the S protein of SARS-CoV-2 (26). We found that the K^b^/S525 tetramers generated by the NIH tetramer facility only stained CD8 T cells from lungs of SARS-CoV-2–infected mice (Fig. 1*B*) and not IAV-infected mice (*SI Appendix*, Fig. S1). Concomitant with viral control in lungs, high frequencies of K^b^/S525-specific CD8 T cells were detected in lungs of SARS-CoV-2 mice at day 10 postinfection (Fig. 1 *B* and *C*). Notably, there was a drastic 116.7-fold contraction of S525-specific CD8 T cells in the lung between days 10 and 38 postinfection.

### Strong Effector CD8 and CD4 T Cell Responses to Mucosal and Parenteral SARS-CoV-2 S Protein–Based Subunit Vaccines.

**Fig. 2.**
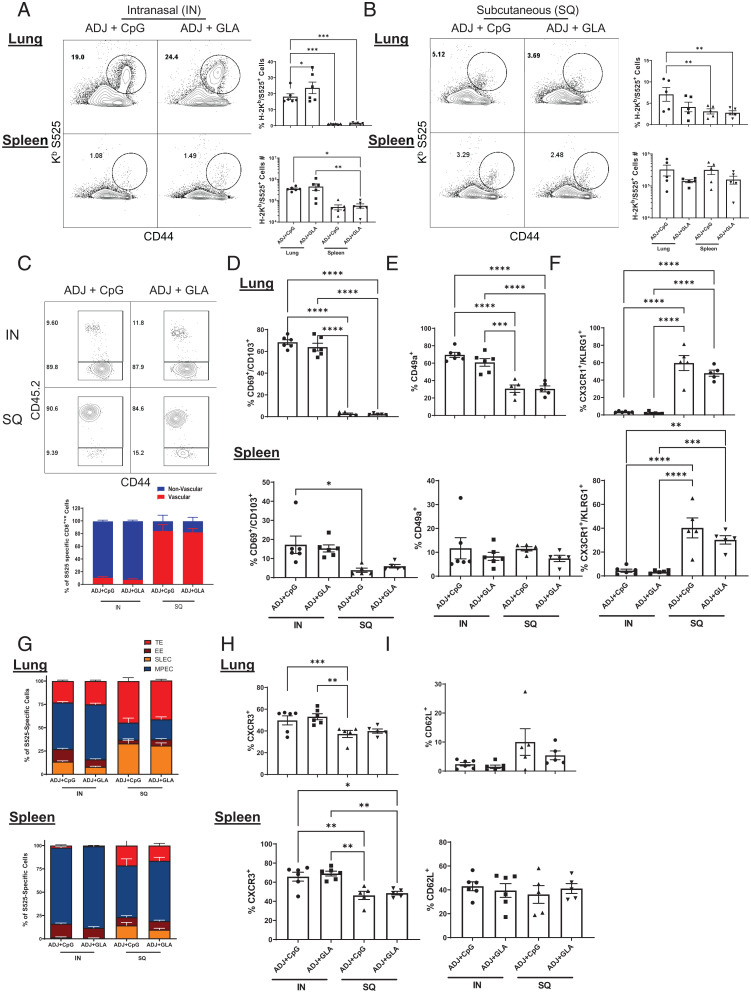
Spike-specific mucosal and systemic effector CD8 T cell responses to adjuvanted vaccines. C57BL/6 mice were IN or SQ vaccinated twice (21 d apart) with recombinant SARS-CoV-2 coronavirus S protein formulated in ADJ (5%) and CpG (5 μg) or GLA (5 μg). To identify circulating/vascular cells in the lungs, mice were injected intravenously with labeled anti-CD45.2 antibodies, 3 min prior to killing (CD45.2^−^, nonvascular; CD45.2^+^, vascular). At day 8 postimmunization, single-cell suspensions from lungs and spleens were stained with viability dye, followed by H2-K^b^/S525 tetramers in combination with anti-CD4, CD8, CD44, CD69, CD103, CD49a, CX3CR1, KLRG1, CD127, CXCR3, and CD62L antibodies. (*A* and *B*) FACS plots show numbers and percentages of S525 tetramer-binding cells among CD8 T cells from IN-vaccinated mice (*A*) and SQ-vaccinated mice (*B*). (*C*) Relative proportions of vascular (CD45.2^+^) and nonvascular (CD45.2^−^) cells among S525-specific CD8 T cells. (*D*–*I*) Percentages of CD103, CD69, CD49a, CX3CR1, KLRG1, CD127, CXCR3, and CD62L^+^ cells among S525-specific CD8 T cells in lungs and spleen. Data represent one of two independent experiments. Asterisks indicate significance at *P < 0.05, **P < 0.005, ***P < 0.0005, and ****P < 0.00005. Data in each graph indicate mean ± SEM.

We have previously reported that mucosal immunization with a subunit protein formulated in a combination adjuvant (carbomer-based nanoemulsion adjuvant Adjuplex [ADJ] with Toll-like receptor [TLR]-4 agonist glucopyranosyl lipid A [GLA] or TLR-9 agonist [CpG]) elicited a multifaceted T cell response in lungs/airways and provided effective heterosubtypic immunity to IAV (23). In contrast, systemic T cell responses induced by parenteral immunization with the ADJ-based subunit vaccine failed to protect against IAV in lungs (22). To evaluate this adjuvanted vaccine approach in the SARS-CoV-2 model, we vaccinated cohorts of C57BL/6 mice twice (3-wk apart) with recombinant S protein (USA-WA1/2020 strain) formulated in ADJ+GLA or ADJ+CpG by the intranasal (IN) or subcutaneous (SQ) routes. At day 8 after booster vaccination, we compared S protein-specific CD8 T cell responses in the respiratory tract and spleen (Fig. 2 and *SI Appendix*, Fig. S2). IN vaccination with ADJ+GLA or ADJ+CpG elicited strong CD8 T cell responses in lungs, but stimulated a less potent CD8 T cell response in spleens (Fig. 2*A*). SQ vaccination with ADJ+GLA and ADJ+CpG evoked comparable CD8 T cell responses in lungs and spleens (Fig. 2*B*). Intravascular staining of CD8 T cells demonstrated that IN vaccination led to the development of nonvascular (parenchymal) and mucosally imprinted (CD69^+^CD103^+^CD49a^+^) CXCR3^+^ CD8 T cells in lungs (Fig. 2 *C*–*E* and *H* and *SI Appendix*, Fig. S2). In contrast, SQ vaccination did not result in mucosal imprinting but led to development of vascular/systemic circulating KLRG-1^HI^CX3CR1^HI^ effector CD8 T cells in lungs and spleens (Fig. 2 *C* and *F*). Furthermore, we analyzed vaccine-induced differentiation of short-lived effector cells (SLECs; KLRG-1^HI^CD127^LO^), memory precursor effector cells (MPECs; KLRG-1^LO^CD127^HI^), transition effectors (TE; KLRG-1^HI^CD127^HI^), and early effectors (EE; KLRG-1^LO^CD127^LO^) (Fig. 2*G* and *SI Appendix*, Fig. S2*F*). A major proportion of effector CD8 T cells induced by IN vaccination in both lungs and spleens were MPECs, while SQ vaccination induced a mixed differentiation of SLECs, MPECs, EEs, and TEs in lungs and spleens (Fig. 2*G* and *SI Appendix*, Fig. S2*F*). In sum, IN vaccination nurtured the development of parenchymal T_RM_-like CD8 T cells in the respiratory tract, and SQ vaccination promoted the development of splenic and predominantly vascular (i.e., systemic or circulating) effector CD8 T cells in lungs.

**Fig. 3.**
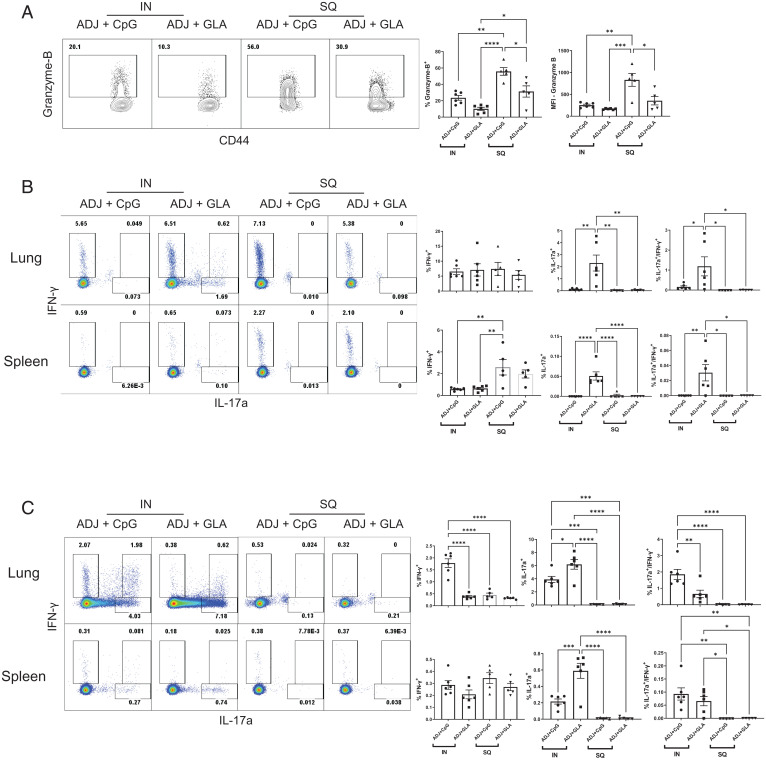
Functional polarization of spike-specific effector CD4 and CD8 T Cells induced by adjuvanted vaccines. C57BL/6 mice were immunized as described in Fig. 2. (*A*) On day 8 after booster vaccination, lung cells were stained with anti-CD8, K^b^/S525 tetramers and antigranzyme B antibodies directly ex vivo. (*B* and *C*) Cytokine production by CD8 and CD4 T cells. On day 8 after booster vaccination, lung cells or splenocytes from were cultured with the S525 peptide for CD8 T cells or SARS CoV-2 S protein peptide pool (encompassing amino acids of S protein from 1 to 451) for CD4 T cells, recombinant IL-2, and Brefeldin A for 5 h. (*B*) S525-specific cytokine-producing CD8 T cells. FACS plots in *B* are gated on CD8 T cells and data are the percentages of IFN-γ– and IL-17a–producing cells among the gated population. (*C*) S peptide pool-specific cytokine-producing CD4 T cells. FACS plots are gated on CD4 T cells and data are the percentages of IFN-γ– and IL-17a–producing cells among the gated population. Data represent one of two independent experiments. Asterisks indicate significance at *P < 0.05, **P < 0.005, ***P < 0.0005, and ****P < 0.00005. Data in each graph indicate mean ± SEM.

Next, we compared the effect of adjuvant and the route of vaccination on the functional polarization (T1/T2/T17) of effector CD8 and CD4 T cells in lungs and spleen (Fig. 3). Fig. 3*A* shows that SQ vaccination (especially with ADJ+CpG) but not IN vaccination strongly promoted the differentiation of granzyme B^+^ effector CD8 T cells. In the lungs, the percentages of interferon (IFN)-γ–producing CD8 T cells were comparable between groups, but IL-17–producing CD8 T cells were induced only in the lungs of ADJ+GLA mice (Fig. 3*B*). Furthermore, those lung effector CD8 T cells that were induced by IN ADJ+CpG or ADJ+GLA were polyfunctional (coproduced IFN-γ, tumor necrosis factor [TNF], and IL-2), as compared to those induced by SQ vaccination (*SI Appendix*, Fig. S3). In spleen, frequencies of IFN-γ–producing CD8 T cells were higher for the SQ groups, but IL-17–producing CD8 T cells were only induced in the ADJ+GLA mice. IN vaccination with ADJ+CpG and ADJ+GLA strongly induced IFN-γ and IL-17–producing CD4 T cells in lungs, and SQ vaccination poorly stimulated cytokine-producing CD4 T cells in spleen or lungs (Fig. 3*C*). Taken together, data in Fig. 3 suggest that 1) ADJ+CpG stimulated a T_C_1 response by both IN and SQ routes, while IN but not SQ ADJ+GLA promoted a mixed T_C_1/T_C_17 response in lungs and spleen; and 2) IN ADJ+GLA and ADJ+CpG stimulated a mixed T_H_1/T_H_17 response in spleen and lungs.

### Humoral Immunity and Memory CD8 and CD4 T Cell Responses to Mucosal and Parenteral SARS-CoV-2 S Protein–Based Subunit Vaccines.

**Fig. 4.**
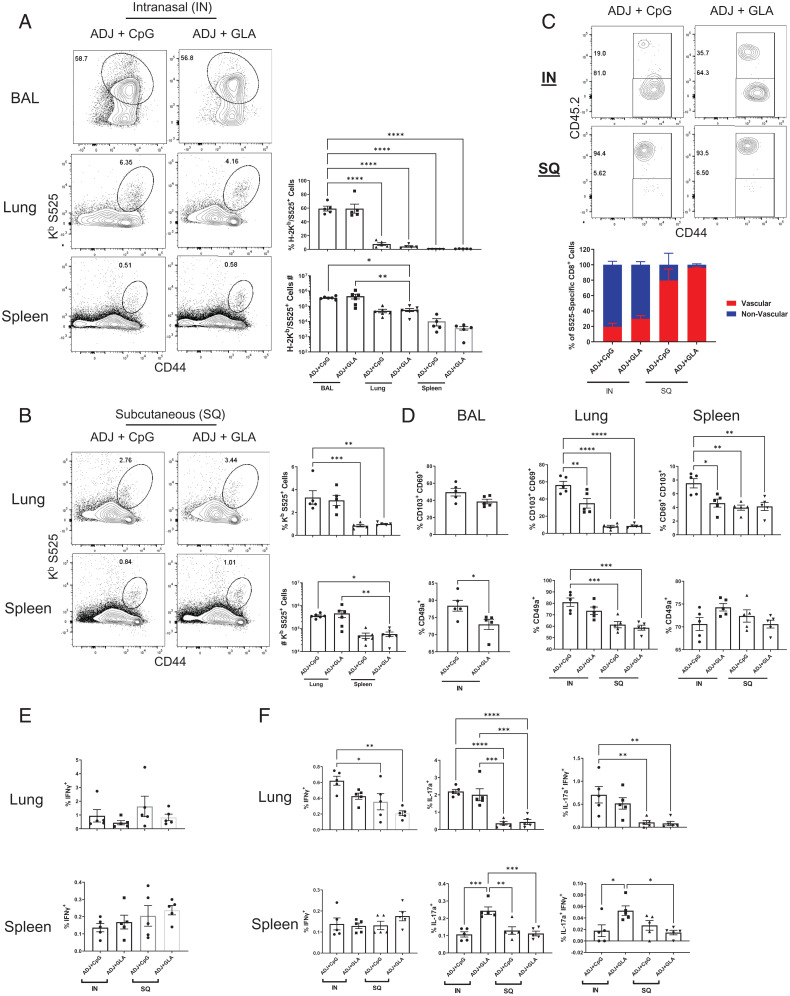
Vaccine-induced mucosal and systemic CD8 T cell memory to S protein. C57BL/6 mice were vaccinated twice IN or SQ with SARS-CoV-2 S protein formulated in ADJ+GLA or ADJ+CpG, as described in Fig. 2. At 44 d after booster vaccination, S525-specific memory CD8 T cells were characterized in airways (BAL), lungs, and spleen. Mice were injected intravenously with PE-labeled anti-CD45.2 antibodies prior to killing, as described in Fig. 2. Single-cell suspensions from various tissues were stained with viability dye, followed by H2-K^b^/S525 tetramers, in combination with anti-CD4, CD8, CD44, CD69, CD103, and CD49a antibodies. (*A*) FACS plots display percentages of H-2K^b^/S525 tetramer-binding cells among CD8 T cells in IN (*A*) or SQ (*B*)-vaccinated mice. (*C*) Percentages of vascular (CD45.2^+^) and nonvascular cells (CD45.2^−^) among S525-specific CD8 T cells. (*D*) Percentages of CD103^hi^, CD69^hi^, CD49a^+^ cells among S525-specific CD8 T cells in airways, lung, and spleen. Lung cells or splenocytes from vaccinated mice were stimulated with the S525 peptide or SARS-CoV-2 S protein peptide pool (encompassing from amino acids 1 to 451), recombinant IL-2 and Brefeldin A for 5 h. Percentages of cytokine-producing cells among CD8 and CD4 T cells were quantified by intracellular cytokine staining. (*E*) Percentages of S525-specific IFN-γ–producing CD8 T cells. (*F*) Percentages of IFN-γ– and IL-17a–producing cells among CD4 T cells upon stimulation with S protein peptide pool. Data represent two independent experiments. Asterisks indicate significance at *P < 0.05, **P < 0.005, ***P < 0.0005, and ****P < 0.00005. Data in each graph indicate mean ± SEM.

Next, we quantified and characterized memory T cells in respiratory tract and spleen at 44 d after the booster vaccination (Fig. 4 *A* and *B*). IN vaccines formulated in ADJ+CpG or ADJ+GLA elicited robust frequencies of memory CD8 T cells in bronco-alveolar lavage (BAL)/airways (∼50% of CD8 T cells) and lungs (5% of CD8 T cells), and lower frequencies in spleen (Fig. 4*A*). The majority of lung memory CD8 T cells elicited by IN vaccines resided in the parenchymal/nonvascular compartment (Fig. 4*C*). In general, 40 to 80% of IN vaccine-elicited memory CD8 T cells in airways and lungs expressed T_RM_ markers CD69 and CD103 (Fig. 4*D* and *SI Appendix*, Fig. S4*A*). Expression of CD49a appeared to be at higher levels in airway and lung memory CD8 T cells elicited by the IN ADJ+CpG group (Fig. 4*D* and *SI Appendix*, Fig. S4*B*). SQ vaccination with ADJ+CpG or ADJ+GLA stimulated similar numbers of memory CD8 T cells in lungs compared to spleen, but these cells were predominantly vascular and failed to express T_RM_ markers CD69 and CD103 (Fig. 4 *B* and *D* and *SI Appendix*, Fig. S4). Regardless of the vaccine adjuvant or the route of vaccination, memory CD8 and CD4 T cells in lungs and spleen produced IFN-γ (Fig. 4 *E* and *F*). Only lung memory CD4 T cells elicited by IN ADJ+CpG or ADJ+GLA produced IL-17a, or coproduced IL-17a and IFN-γ (Fig. 4*F*). Taken together, data in Fig. 4 and *SI Appendix*, Fig. S4 showed that IN vaccination elicited lung/airway T1/T17 T_RM_s and SQ vaccination instead elicited T1 systemic/vascular memory T cells.

**Fig. 5.**
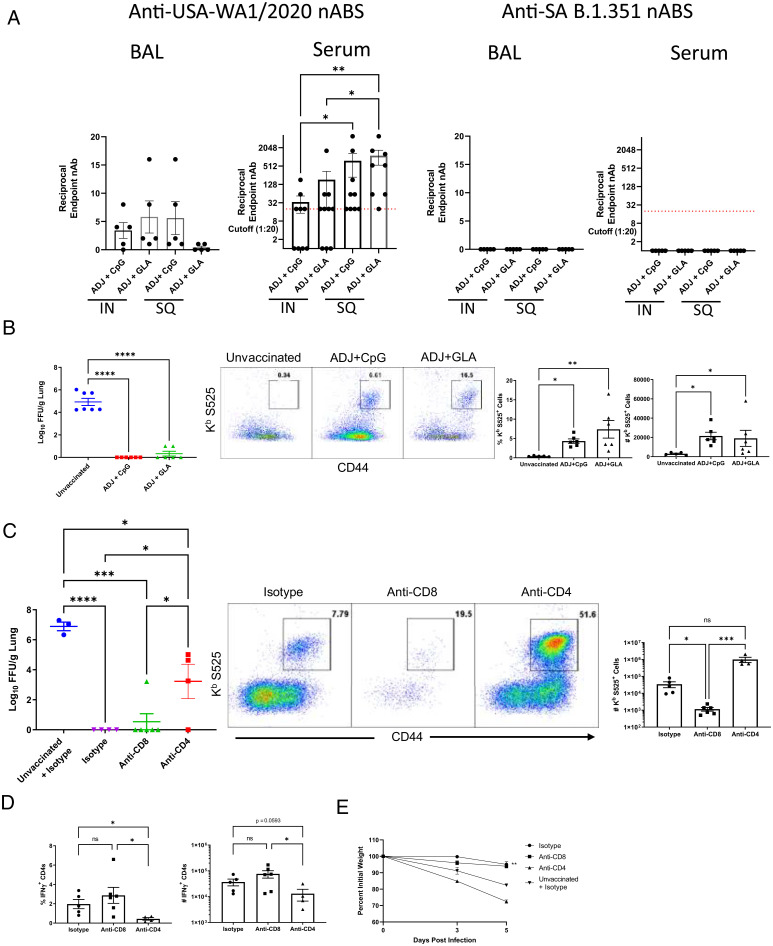
Vaccine-induced virus-neutralizing antibodies and protective immunity to homologous WA Strain of SARS-CoV-2. Six- to 8-wk-old K18-ACE2tg mice were vaccinated twice IN or SQ with SARS-Co-V-2 S protein formulated in ADJ+GLA or ADJ+CpG. At 44 d after booster vaccination, BAL (undiluted to 1:80 dilution) and serum samples (starting at 1:20 dilution) were tested for SARS-COV-2–neutralizing capacity by a microneutralization assay. Virus neutralization was tested against two SARS-CoV-2 variants: USA-WA1/2020 (WA strain) and B.1.351 (SA variant). (*A*) Endpoint neutralization titer is shown against the indicated SARS-CoV-2 viruses. (*B*) Mice were vaccinated by the IN route as above, at 50 d after boost, when mice were ∼17 wk of age, they were challenged with the WA strain of SARS-CoV-2 virus; unvaccinated mice were challenged as controls. On day 5 after viral challenge, viral titers and S525-specific CD8 T cells were quantified in the lungs. (*B*) Viral titers in lungs and recall S525-specific CD8 T cell responses. Graphs show numbers and percentages of tetramer-binding cells gated on total CD8 T cells in lungs. (*C*) Mice were vaccinated with ADJ+GLA by the IN route as above and were treated with isotype control antibodies, anti-CD4 antibodies (during primary and booster vaccination) or anti-CD8 antibodies (prior to viral challenge). At 55 d postbooster vaccination when mice were ∼18 wk of age, they were challenged with the WA strain of SARS-CoV-2 virus. (*C*) Figures show viral titers and recall S525-specific CD8 T cell responses in lungs. Graphs show numbers of tetramer-binding cells gated on total CD8 T cells in lungs. (*D*) Frequencies and total numbers of IFN-γ–producing CD4 T cells in lungs were quantified by intracellular cytokine staining after stimulation with S protein peptide pool as above. (*E*) Graph showing weight loss as a percent of initial starting weight over days following infection. Asterisks indicate significance at *P < 0.05, **P < 0.005, ***P < 0.0005, and ****P < 0.00005; ns, not significant . Data in each graph indicate mean ± SEM.

To determine if subunit vaccine elicited antibody responses to the SARS-CoV-2 S protein, serum and BAL collected from mice at 44 d postbooster vaccination were tested for neutralizing antibodies against the homologous USA-WA1/2020 strain of SARS-CoV-2 (Fig. 5*A*). Serum samples from mice vaccinated by the SQ route had high average titers of virus-neutralizing antibodies. However, serum samples from 4 of 9 and 3 of 10 mice, respectively, from each of the IN ADJ+CpG and IN ADJ+GLA groups had undetectable virus-neutralizing activity at the lowest dilution tested (1:20). Except for one mouse in the ADJ+CpG group, all mice in the two IN groups had detectable virus-neutralizing activity in BAL samples when tested either undiluted or at dilutions up to 1:16. Interestingly, all mice in the SQ ADJ+CpG had neutralizing antibodies in the BAL, while three of five mice in the SQ ADJ+GLA had no detectable titers in BAL. Next, we assessed whether antibodies raised against the USA-WA1/2020 S protein would neutralize the B.1.351 variant (South Africa; β-variant) of SARS-CoV-2. Surprisingly, regardless of the route of vaccination, we did not detect virus-neutralizing activity against B.1.351 SARS-CoV-2 in serum or BAL of vaccinated mice (Fig. 5*A*), indicating that our vaccines might have failed to induce antibodies that can neutralize the infectivity of B.1.351 β-variant of SARS-CoV-2, at least in vitro.

### Vaccine-Induced Systemic vs. Mucosal Protective Immunity to Homologous and Variant SARS-CoV-2 Challenge.

To assess whether mucosal vaccination with adjuvanted S protein subunit vaccines afforded protective immunity to COVID-19, we immunized K18-hACE2tg mice IN with SARS-CoV-2 S protein formulated in ADJ+GLA or ADJ+CpG twice at an interval of 3 wk. At 50 d after the booster vaccination, vaccinated and unvaccinated mice were challenged with 5 × 10^4^ PFU of the homologous USA-WA1/2020 strain of SARS-CoV-2. On the fifth day after viral challenge, we quantified SARS-CoV-2 burden (Fig. 5*B*) and recall T cell responses in lungs (Fig. 5*B*). As shown in Fig. 5*B*, lungs of unvaccinated mice contained 4 to 6 log_10_s of SARS-CoV-2, but infectious virus in the lungs of ADJ+GLA- or ADJ+CpG-vaccinated mice ranged from levels that were undetectable to those that were barely above the threshold of detection. Following viral challenge, recall S525-specific CD8 T cell responses in lungs of mice were detected in relatively high frequencies and numbers in vaccinated mice, but not in unvaccinated mice (Fig. 5*B*). We also determined whether vaccines protected against SARS-CoV-2–induced lung pathology following challenge (*SI Appendix*, Fig. S5 *A*–*F*). Lungs of unvaccinated mice showed substantive alveolar lung damage with sparing airways. Alveolar damage was reflected by alveolar thickening with necrosis, edema, and increased numbers of interstitial macrophages and lymphocytes. In contrast, regardless of the vaccine used, lungs of vaccinated mice were protected from SARS-CoV-2–induced alveolar damage. Likewise, unvaccinated mice lost substantive body weight, but mice in the ADJ+CpG group showed no significant weight loss and ADJ+GLA group only experienced transient weight loss (*SI Appendix*, Fig. S5*G*). In our studies, regardless of being vaccinated or not, virally challenged K18-hACE2tg mice did not display neurological symptoms at least until the time of killing (day 5). Thus, IN vaccination with spike protein formulated in either ADJ+GLA or ADJ+CpG conferred effective protection against SARS-CoV-2 challenge.

To increase the rigor of our studies, it was of interest to test the vaccine effectiveness in another mouse model of SARS-CoV-2 infection. To confirm vaccine-induced protective immunity in a different SARS-CoV-2 challenge model and compare the effectiveness of mucosal versus parenteral immunization, we vaccinated cohorts of unmanipulated C57BL/6 mice with S protein formulated in ADJ+GLA or ADJ+CpG either by the IN or the SQ route. At 101 d after immunization, vaccinated and unvaccinated C57BL/6 mice were primed with IN administration of human ACE2-expressing adenovirus (27), and these mice were subsequently challenged with the homologous USA-WA1/2020 strain of SARS-CoV-2. At day 5 after SARS-CoV-2 challenge, regardless of the adjuvant used or the vaccination route, lungs of vaccinated mice contained 4 to 6 log_10_ lower SARS-CoV-2 load, as compared to viral load in lungs of unvaccinated mice (*SIAppendix*, Fig. S6). Thus, adjuvanted subunit vaccines administered mucosally or parenterally conferred effective protection against mucosal challenge with the homologous Washington strain of SARS-CoV-2.

Next, we investigated the immune mechanisms underlying vaccine-induced protection against homologous challenge with the USA-WA1/2020 strain of SARS-CoV-2. First, we ablated the development of protective CD4 T cell–dependent immunity (including antibody, CD8 T cell programming, and CD4 T cell effector mechanisms) by depleting CD4 T cells in a cohort of mice at the time of primary and booster immunization IN with ADJ+GLA. At 50 d after vaccination, we found that serum from all CD4 T cell–depleted mice did not possess detectable neutralizing antibodies to the USA-WA1/2020 strain of SARS-CoV-2. Second, to evaluate the importance of vaccine-elicited memory CD8 T cells in protective immunity to SARS-CoV-2, we depleted CD8 T cells (mucosally and systemically) in a cohort of vaccinated mice just prior to viral challenge. At 55 d after vaccination, cohorts of undepleted, CD4 T cell–depleted, and CD8 T cell–depleted vaccinated mice were challenged with a lethal dose of the USA-WA1/2020 strain of SARS-CoV-2; unvaccinated mice were challenged as controls (Fig. 5*C*). At day 5 after viral challenge, lungs of unvaccinated mice contained high viral burden, but lungs of undepleted vaccinated mice contained undetectable levels of virus, as in Fig. 5*C*. Lungs of vaccinated CD8 T cell–depleted mice contained very few residual CD8 T cells (Fig. 5*C*), but low levels of virus in lungs (Fig. 5*C*), which suggested that depletion of memory CD8 T cells did not compromise vaccine-induced immunity to homologous SARS-CoV-2 challenge.

In striking contrast, mice that were depleted for CD4 T cells during vaccination had significantly higher levels of lung viral burden than in lungs of undepleted and CD8 T cell–depleted mice. Significantly (*P* < 0.05) lower frequencies of IFN-γ−producing CD4 T cells were detected in lungs of mice depleted for CD4 T cells during vaccination, as compared to those in lungs of undepleted and CD8 T cell–depleted mice (Fig. 5*D*). Despite increased lung cellularity in the virally challenged CD4 T cell–depleted mice, the total number of IFN-γ−producing CD4 T cells were ∼70% lower (*P* = 0.0593) than in undepleted mice (Fig. 5*D*). It is worth pointing out that one mouse in the CD4 T cell–depleted group that controlled virus (Fig. 5*C*) also had higher numbers of IFN-γ^+^ CD4 T cells (Fig. 5*D*) but demonstrated weight loss after viral challenge. These findings ascribe a vital role for CD4 T cell–dependent immune mechanisms in vaccine-induced immunity to SARS-CoV-2. Interestingly, however, extremely high frequencies and numbers of S525-specific CD8 T were detected in lungs after challenge in this CD4-depleted group (Fig. 5*C*), indicating there was a robust recall expansion of unhelped S525-specific CD8 T cells, and that these high numbers of CD8 T cells were insufficient to fully compensate for the absence of detectable virus-neutralizing antibodies and memory CD4 T cells. However, it is important to note that it appears that these unhelped virus-specific CD8 T cells in this CD4 depletion group accounted for some degree of viral control, as the level of virus in this cohort was significantly lower than in the unvaccinated group (Fig. 5*C*). Upon viral challenge, weight loss was only evident in unvaccinated and CD4 T cell–depleted groups but not in CD8 T cell–depleted or undepleted groups (Fig. 5*E*). Taken together, these results strongly suggest a primary role for CD4 T cell–dependent mechanisms, including antibodies in vaccine immunity to infection with the homologous USA-WA1/2020 strain of SARS-CoV-2 virus in mice.

**Fig. 6.**
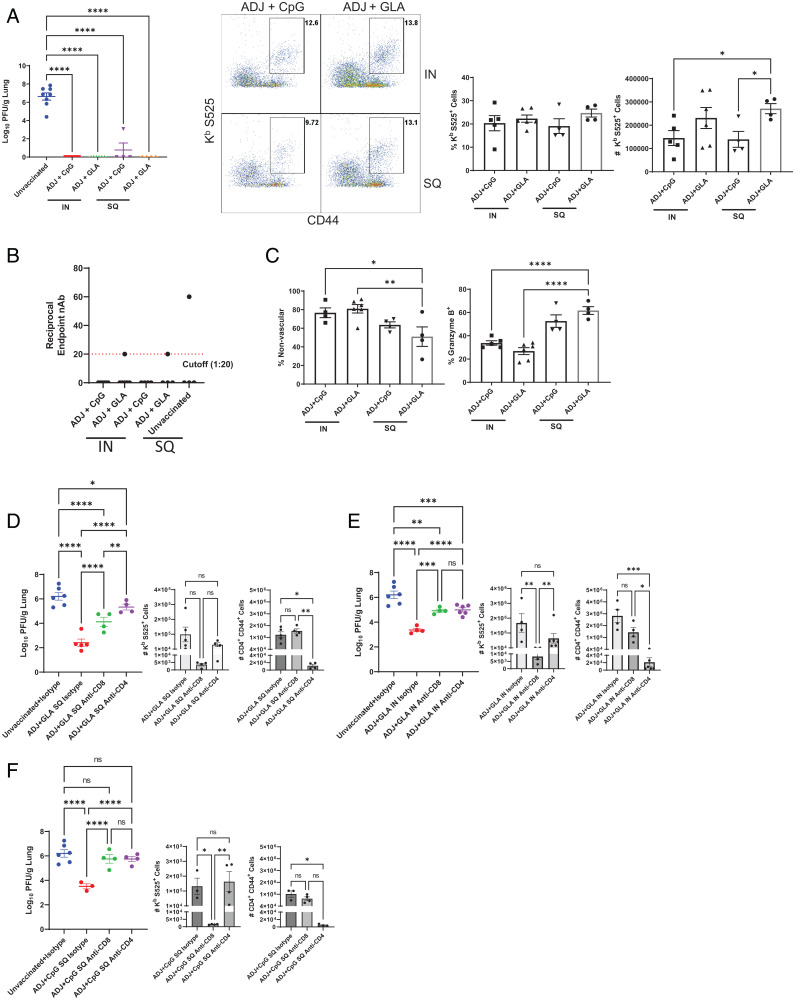
Vaccine-induced protective immunity to the SA variant of SARS-CoV-2 (B.1.351). Cohorts of 6- to 8-wk-old K18-ACE2tg mice were vaccinated twice with S protein of the WA strain of SARS-CoV-2, as described in Fig. 2. At 65 d after booster vaccination, when mice were ∼18 wk of age, they were challenged with the B.1.351 variant of SARS-CoV-2 virus; unvaccinated mice were challenged as controls. (*A*) Viral titers and S525-specific CD8 T cells were quantified in the lungs on day 5 after challenge. Graphs show percentages and total numbers of S525-specific CD8 T cells in lungs. (*B*) Neutralizing antibody titers to the SA B.1.351 variant in serum samples collected from mice at day 5 after challenge upon necropsy. (*C*) Percentages of vascular nonvascular (CD45.2^−^) and granzyme B^+^ cells among S525-specific CD8 T cells. (*D*–*F*) Cohorts of 6-wk-old K18-hACE2tg mice were vaccinated once with USA-WA1/2020 S protein formulated in ADJ+GLA SQ (*D*) or IN (*E*) route, or with ADJ+CpG SQ (*F*). Twenty-one days after vaccination, when mice were ∼9 wk of age, mice were treated IN and intravenously with anti-CD4 or anti-CD8 antibodies before and during challenge with the B.1.351 variant of SARS-CoV-2. Graphs show viral titers, S525-specific CD8^+^ and activated (CD44^+^) CD4 T cells in lungs on day 5 after challenge quantified by staining and flow cytometry, as described above. Asterisks indicate significance at *P < 0.05, **P < 0.005, ***P < 0.0005, and ****P < 0.00005; ns, not significant . Data in each graph indicate mean ± SEM.

Several SARS-CoV-2 viral variants have emerged in the United States and elsewhere after the initial pandemic strain of SARS-CoV-2. There is evidence that some of the viral variants might be more infectious and even less susceptible to neutralization with antibodies raised against the early strains of SARS-CoV-2 (4, 5, 28–31). Our adjuvanted vaccines failed to stimulate detectable levels of B1.351 variant virus-neutralizing antibodies (Fig. 5*A*), and likewise, the currently used mRNA vaccines also elicit weak neutralizing antibody response to the B.1.351 variant in humans (32, 33). Therefore, the ADJ-based combination adjuvant vaccination strategy provided us with a model to explore whether immune mechanisms bereft of virus-neutralizing antibodies can protect against the relatively antibody-resistant B.1.351 variant of SARS-CoV-2. Cohorts of K18-hACE2tg mice were vaccinated with the USA-WA1/2020 S protein formulated in ADJ+GLA or ADJ+CpG by the IN or the SQ route, as above. At 65 d after booster vaccination, we challenged vaccinated and unvaccinated mice with the B.1.351 β-variant of SARS-CoV-2, and lung viral titers were quantified at day 5 after viral challenge. Unvaccinated mice had high viral burden in the lungs in the range of 6 to 8 log_10_s of SARS-CoV-2 (Fig. 6*A*). Strikingly, despite the absence of detectable prechallenge virus-neutralizing antibodies in airways or serum of vaccinated mice, nearly complete control of viral replication was observed in all vaccine groups, regardless of the route of vaccination (Fig. 6*A*). It is noteworthy that, even after viral challenge, virus-neutralizing antibodies were below the level of detection in almost all vaccinated animals, regardless of the vaccination route (Fig. 6*B*).

Taken together, these data suggested that our experimental vaccines provided effective protection to the B.1.351 variant of SARS-CoV-2, in the apparent absence of detectable virus-neutralizing antibodies, implicating a role for T cells in vaccine-elicited protective immunity. Consistent with this idea, S525-specific CD8 T cell recall responses were observed in greater frequencies and numbers in mice challenged with B.1.351 variant (Fig. 6*A*) and were of relatively higher magnitude than recall responses in mice challenged with the USA-WA1/2020 strain (Fig. 5*B*). Although no differences in viral control were observed in IN vs. SQ groups (Fig. 6*A*), IN vaccine groups displayed greater accumulation of effector CD8 T cells in the nonvascular lung tissue, as compared to SQ vaccine groups (Fig. 6*C*). Additionally, higher percentages of effector CD8 T cells in SQ groups contained granzyme B than in IN groups (Fig. 6*C*). Taken together, these data indicated that ADJ+GLA or ADJ+CpG formulated with subunit S protein can elicit broad protective immunity to SARS-CoV-2 by both parenteral and mucosal vaccination.

To assess whether enhanced viral control in vaccinated mice was also associated with protection from SARS-CoV-2–induced lung damage, we performed histopathological analysis of lungs following viral challenge (*SI Appendix*, Fig. S7 *A*–*G*). Lungs of virally challenged unvaccinated mice displayed alveolar necrosis, alveolar wall thickening, edema, and alveolar histiocytosis with type II pneumocyte hyperplasia. Vaccines largely protected lungs from alveolar damage but displayed prominent peribronchial and perivascular lymphoid infiltrations. The lungs of 4 of 11 mice only in the ADJ+GLA IN group exhibited severe multifocal nonsuppurative interstitial pneumonia and alveolar damage, severe histiocytosis, and type II pneumocyte hyperplasia, affecting up to 80% of the most severely affected lung sections (*SI Appendix*, Fig. S7*F*). Consistent with effective viral control and limited lung pathology, mice in all vaccinated groups were protected from weight loss following viral challenge (*SI Appendix*, Fig. S7*H*). No overt neurological symptoms were observed in any mice for at least until 5 d after viral challenge. Thus, all vaccines tested in this study largely protected against weight loss and lung pathology, induced by the B.1.351 β-variant of SARS-CoV-2.

To test if protection elicited by these vaccines was durable by both IN and SQ routes, cohorts of K18-hACE2tg mice were vaccinated IN or SQ with the USA-WA1/2020 spike protein formulated in ADJ+GLA, as above. At 140 d after booster vaccination, we challenged vaccinated and unvaccinated mice with the B.1.351 β-variant of SARS-CoV-2, and lung viral titers were quantified at day 5 after viral challenge. Vaccination by both IN and SQ routes led to a significant reduction in lung viral titers, protection from weight loss, and substantial S525-specific CD8^+^ T cell recall in lungs of mice (*SI Appendix*, Fig. S8). Serum samples taken at day 5 postchallenge from all vaccinated and unvaccinated animals were tested for neutralizing activity to the B.1.351 variant, and no neutralization was observed at the limit of detection (1:20) in any sample.

Next, it was of interest to assess the relative roles of CD4 and CD8 T cells in protective immunity to the B.1.351 variant of SARS-CoV-2. To this end, we vaccinated cohorts of K18-hACE2tg mice with USA-WA1/2020 S protein formulated in ADJ+GLA (SQ or IN route) or ADJ+CpG (SQ route) as above. Twenty-one days after vaccination, mice were treated IN and intravenously with anti-CD4 or anti-CD8 antibodies and challenged with B.1.351 β variant of SARS-CoV-2. A single dose of vaccination (SQ or IN) with the S protein formulated in ADJ+GLA or ADJ+CpG effectively reduced viral titers in the lungs by more than 99%; viral titers in lungs of isotype antibody-treated vaccinated mice were 2∼3 log_10_ lower, as compared to those in lungs of unvaccinated mice (Fig. 6 *D*–*F*). Depletion of CD4 or CD8 T cells in vaccinated mice resulted in significantly greater (*P* < 0.05) viral titers in the lungs, as compared to those in vaccinated isotype antibody-treated mice (Fig. 6 *D*–*F*). Depletion efficacy was confirmed by quantifying numbers of CD4^+^ CD44^+^ or S525-specific CD8 T cells in lungs of mice; numbers of antigen specific CD8^+^ or activated CD4 T cells were significantly reduced respective to antibody treatment (Fig. 6 *D*–*F*). These data suggest that memory CD4 and helped CD8 T cells contribute to protection against B.1.351 β-variant of SARS-CoV-2 in the lungs.

It should be noted that the B.1.351 β-variant of SARS-CoV-2 readily replicates to high titers in lungs of unmanipulated C57BL/6 mice (34). Next, we assessed whether adjuvanted vaccines also protected against the B.1.351 β-variant of SARS-CoV-2 in unmanipulated C57BL/6 mice in a T cell–dependent manner. C57BL/6 mice were vaccinated twice IN with S protein (from USA-WA1/2020 strain) formulated in ADJ+GLA. At 154 d after vaccination, mice were depleted of mucosal and systemic CD4 or CD8 T cells by antibody treatment and challenged with B.1.351 β-variant of SARS-CoV-2. While vaccinated mice treated with isotype control antibodies effectively controlled lung virus replication, lungs of unvaccinated mice contained significantly higher (*P* < 0.05) viral burden (*SI Appendix*, Fig. S9). Strikingly, depletion of CD4 T cells or CD8 T cells led to significant increase (*P* < 0.05) in viral load in lungs, as compared to mice treated with isotype control antibodies (*SI Appendix*, Fig. S9).

## Discussion

According to the current axiom, high levels of virus-neutralizing antibodies at portals of viral entry can often confer sterilizing immunity to infections. On the other hand, memory T cells may not provide sterilizing immunity, but curtail viral infections and protect against disease. Hence, it is desirable to develop vaccines that engender both humoral and T cell memory and afford effective protection through two complementary layers of immunity. With the rapid emergence of highly contagious SARS-CoV-2 variants, such as OMICRON that evade neutralizing antibodies (35–37), there is an urgent need for developing broadly protective vaccines against COVID-19. In this context, there exists a knowledge gap of whether memory T cells can confer immunity to SARS-CoV-2, especially under conditions where virus effectively evades neutralizing antibodies. In this study, using experimental subunit vaccines, we investigated the tenets of mucosal/resident vs. systemic/migratory T cell immunity in protection against lethal mucosal challenge with the β-variant of SARS-CoV-2 that evades virus-neutralizing antibodies.

Although virus-neutralizing antibodies are front and center to immunity induced by the currently used SARS-CoV-2 vaccines, there is emerging argument that complementary T cell immunity would be paramount for broad protective immunity to newly emerging viral variants (4, 5, 7, 9, 25, 30, 38). Using the ADJ-based adjuvant system, we questioned whether the tenets of pulmonary T cell immunity to influenza and SARS-CoV-2 are different. Our studies confirmed that mucosal immunization elicited airway/lung T_RM_s and antibodies in airways, while parenteral immunization induced circulating memory CD4 and CD8 T cells and antibodies. The presence of mucosal or systemic humoral and T cell immunity effectively protects against the homologous Washington strain of SARS-CoV-2; CD4 T cell–dependent immunity (antibodies and CD4 T cells) but not CD8 T cells were nonredundant for vaccine immunity to the Washington strain of SARS-CoV-2. Notably, in our studies, unhelped memory CD8 T cells in CD4 T cell–depleted mice expanded but failed to protect against the homologous Washington strain of SARS-CoV-2. This finding is consistent with a role for memory CD4 T cells in viral control and in programming protective CD8 T cell memory (39), but incisive studies are warranted to assess whether helped memory CD8 T cells can protect against homologous SARS-CoV-2 challenge, in the absence of neutralizing antibodies and robust CD4 T cell responses.

Most strikingly, even in the absence of detectable virus-neutralizing antibodies in the respiratory tract or serum, mucosal or systemic memory T cells, along with nonneutralizing antibodies, likely conferred effective protective immunity to the South African SARS-CoV-2 β-variant B.1.351. It should be noted that, unlike a lesser role for vaccine-elicited memory CD8 T cells in vaccine immunity to the Washington strain, vaccine-elicited pulmonary memory CD4 and CD8 T cells play nonredundant roles in mediating protection against the SARS-CoV-2 variant B.1.351. The differences in the contributions of CD8 T cells to protection against the two strains might be linked to the primacy of virus-neutralizing antibodies in protection against the homologous Washington strain but not the B.1.351 variant of SARS-CoV-2. There is building consensus that T cell-based protective immunity to IAV is mediated by airway and lung T_RM_s, and our previous work show that only mucosally administered vaccines, but not parenteral vaccines, induce such T_RM_s and protect against influenza (16–19, 22). We have also shown that combination nanoemulsion adjuvants containing ADJ and TLR agonists GLA or CpG induced high numbers of lung CD8 and CD4 T_RM_s and protected against multiple strains of IAV (23). Unlike T_RM_-centric protection against IAV, either mucosal or parenteral vaccinations protect against the β-variant of SARS-CoV-2, which suggest that both T_RM_s and systemic migratory memory T cells can protect against SARS-CoV-2. Likewise, systemic migratory T cell memory controls mucosal challenge with vaccinia virus (40). Taken together, these findings highlight the differences in tenets of T cell–dependent protective immunity to respiratory infections with viruses, such as influenza virus, SARS-CoV-2, and poxvirus, and demonstrate the relative importance of antibodies and T cells in immune prophylaxis against SARS-CoV-2 variants.

Additionally, we found that both memory CD4 and CD8 T cells contribute to protection against the SARS-CoV-2 variant B.1.351, but the exact mechanisms remain unknown. While memory CD8 T cells can mediate viral control by MHC I-restricted cellular cytotoxicity, the role of noncytolytic viral control mechanisms including the production of cytokines, such as IFN-γ or IL-17, cannot be excluded. Memory CD4 T cells can potentially contribute to viral control directly by producing antiviral cytokines or indirectly by augmenting memory B cell-dependent anamnestic antibody responses (41) or by promoting the activation and trafficking of memory CD8 T cells. However, we did not detect anamnestic neutralizing antibody responses in the serum following challenge with variant B.1.351, nor did we find lower CTL numbers in the lungs of CD4 T cell-depleted virally challenged mice. Pertaining to the role of T1 vs. T17 immunity in protection, only ADJ+GLA IN vaccine elicited mixed populations of T1 and T17 CD4 and CD8 T cells; ADJ+CpG IN only induced T_H_17 but not T_C_17 memory T cells. Regardless of the elicitation of T1 and T17 immunity, we did not observe detectable differences in SARS-CoV-2 control in lungs between vaccines. However, only in a subset of ADJ+GLA IN mice challenged with β-variant B.1.351 did lungs display severe histological changes, and it will be important to determine whether T17 immunity underlies this lung pathology. In sum, further studies are warranted to understand the mechanisms underlying the memory CD4 and CD8 T cell–dependent control of SARS-CoV-2.

The currently used SARS-CoV-2 mRNA vaccines continue to provide substantive protection against severe disease, despite the emergence of SARS-CoV-2 variants that have grown increasingly resistant to neutralization by vaccine- or infection-elicited antibodies. In this context, it is noteworthy that SARS-CoV-2 mRNA vaccines induce both antibodies and cross-reactive memory T cells (42, 43). The specific role of memory T cells induced by SARS-CoV-2 mRNA vaccines in protection of humans against SARS-CoV-2 infection and severe disease is unclear. Our studies clearly show that systemic or mucosal memory T cells can mediate effective viral control upon challenge with the relatively antibody-resistant B.1.351 variant of SARS-CoV-2. Thus, it is possible that cross-reactive memory T cells induced by SARS-CoV-2 mRNA vaccines might protect against severe disease induced by the DELTA and the OMICRON variants.

In this study, using an experimental subunit vaccine consisting of SARS-CoV-2 S protein formulated in a nanoemulsion/TLR agonist-based combination adjuvant administered mucosally or parenterally, we probed the fundamental tenets of T cell immunity to SARS-CoV-2 variants that differ in their abilities to evade antibody-based immunity. We ascribe key nonredundant roles for lung-resident and systemic migratory memory CD4/CD8 T cells elicited by mucosal or parenteral immunizations, respectively, in limiting lung replication of SARS-CoV-2 variant that evade antibody-based immune defense. These key findings are expected to have significant implications in the development of broadly protective T cell–based vaccines and cellular immunotherapy against COVID-19.

## Methods

### Experimental Animals.

Seven- to 12-wk-old C57BL/6J (B6) were purchased from restricted-access specific pathogen-free (SPF) mouse breeding colonies at the University of Wisconsin–Madison Breeding Core Facility. K18-hACE2 (Stock number: 034860) mice were purchased from Jackson Laboratory or bred in the above-mentioned SPF facility. All experiments were reviewed and approved by the University of Wisconsin School of Veterinary Medicine Animal Care and Use Committee.

### Reagents.

Reagents used in these studies are listed in *SI Appendix*, *Supplemental Methods* and Table S1.

### Vaccination.

All vaccinations were administered subcutaneously to the tail base or IN to anesthetized mice in 50 μL saline with 10 μg SARS-CoV-2 (2019-nCoV) Spike S1+S2 ECD-His Recombinant Protein (S protein) with the following adjuvants: ADJ (5%) + CpG (5 μg) or ADJ (5%) + GLA (5 μg). Mice were vaccinated twice at an interval of 3 wk.

### Tissue Processing and Flow Cytometry and Ex Vivo Cytokine Analysis.

Spleens and lungs were processed into single-cell suspensions and stained for cellular factors, as previously described (23) and in *SI Appendix*, *Supplemental Methods*.

### Cells and Viruses.

SARS-CoV-2 USA-WA1/2020 (WA strain) and hCoV-19/South Africa/KRISP-EC-K005321/2020 (SA strain) viruses used in these studies were obtained from BEI resources (NR-52281 and NR-55282, respectively) and were cultured as described in *SI Appendix*, *Supplemental Methods*.

### Viral Challenge.

To induce a sublethal SARS-CoV-2 infection, mice were infected with 100 PFU, and lethal doses were given at 5 × 10^4^ PFU for both WA and SA strains by the IN route. For PR8/H1N1 sublethal infection, doses were given at 50 PFU by the IN route. To assess the role of CD4 T cells and CD8 T cells in protective immunity, mice were administered 200 μg of anti-CD4 (Bio X Cell, Clone: GK1.5) or anti-CD8 antibodies (Bio X Cell; Clone 2.43) intravenously and IN at days −5. −3, −1 and 1, 3, and 5, relative to vaccination or challenge as indicated. For challenge studies using ACE2-expressing adenovirus (Ad5-hAce2), mice were administered 7.75 × 10^7^ PFU of Ad5-hAce2 given via IN instillation under isoflurane anesthesia in 50 μL saline. Four days after sensitization, mice were challenged with 1 × 10^5^ PFU of the SARS-CoV-2 WA strain by the IN route.

### Statistical Analyses.

Statistical analyses were performed using GraphPad software 9.0. Comparisons were made using one-way ordinary ANOVA test with multiple comparisons, significance at *P < 0.05, **P < 0.005, ***P < 0.0005, and ****P < 0.00005. Data in each graph indicate mean ± SEM.

## Supplementary Material

Supplementary File

## Data Availability

All study data are included in the main text and *SI Appendix*.

## References

[1] E. Dong, H. Du, L. Gardner, An interactive web-based dashboard to track COVID-19 in real time. Lancet Infect. Dis. 20, 533–534 (2020). 32087114 10.1016/S1473-3099(20)30120-1 PMC7159018

[2] P. Wang , Increased resistance of SARS-CoV-2 variant P.1 to antibody neutralization. Cell Host Microbe 29, 747–751.e4 (2021). 33887205 10.1016/j.chom.2021.04.007 PMC8053237

[3] P. Supasa , Reduced neutralization of SARS-CoV-2 B.1.1.7 variant by convalescent and vaccine sera. Cell 184, 2201–2211.e7 (2021). 33743891 10.1016/j.cell.2021.02.033 PMC7891044

[4] W. Dejnirattisai , Reduced neutralisation of SARS-CoV-2 omicron B.1.1.529 variant by post-immunisation serum. Lancet 399, 234–236 (2021). 34942101 10.1016/S0140-6736(21)02844-0 PMC8687667

[5] M. Kozlov, Omicron overpowers key COVID antibody treatments in early tests. Nature, 10.1038/d41586-021-03829-0 (2021). 34937889

[6] D. S. Khoury , Neutralizing antibody levels are highly predictive of immune protection from symptomatic SARS-CoV-2 infection. Nat. Med. 27, 1205–1211 (2021). 34002089 10.1038/s41591-021-01377-8

[7] A. D. Redd , CD8+ T cell responses in COVID-19 convalescent individuals target conserved epitopes from multiple prominent SARS-CoV-2 circulating variants. Open Forum Infect. Dis. 8, ofab143 (2021). 34322559 10.1093/ofid/ofab143 PMC8083629

[8] H. Kared , SARS-CoV-2-specific CD8+ T cell responses in convalescent COVID-19 individuals. J. Clin. Invest. 131, e145476 (2021). 10.1172/JCI145476 PMC7919723 33427749

[9] A. D. Redd , Minimal cross-over between mutations associated with Omicron variant of SARS-CoV-2 and CD8+ T cell epitopes identified in COVID-19 convalescent individuals. *bioRxiv* [Preprint] (2021). https://www.biorxiv.org/content/10.1101/2021.12.06.471446v1 (Accessed 16 February 2022). 10.1128/mbio.03617-21 PMC8941890 35229637

[10] Z. Chen, E. John Wherry, T cell responses in patients with COVID-19. Nat. Rev. Immunol. 20, 529–536 (2020). 32728222 10.1038/s41577-020-0402-6 PMC7389156

[11] J. M. Dan , Immunological memory to SARS-CoV-2 assessed for up to 8 months after infection. Science 371, eabf4063 (2021). 33408181 10.1126/science.abf4063 PMC7919858

[12] A. Sette, S. Crotty, Adaptive immunity to SARS-CoV-2 and COVID-19. Cell 184, 861–880 (2021). 33497610 10.1016/j.cell.2021.01.007 PMC7803150

[13] D. L. Farber, N. A. Yudanin, N. P. Restifo, Human memory T cells: Generation, compartmentalization and homeostasis. Nat. Rev. Immunol. 14, 24–35 (2014). 24336101 10.1038/nri3567 PMC4032067

[14] S. C. Jameson, D. Masopust, Understanding subset diversity in T cell memory. Immunity 48, 214–226 (2018). 29466754 10.1016/j.immuni.2018.02.010 PMC5863745

[15] D. Masopust, A. G. Soerens, Tissue-resident T cells and other resident leukocytes. Annu. Rev. Immunol. 37, 521–546 (2019). 30726153 10.1146/annurev-immunol-042617-053214 PMC7175802

[16] B. Slütter, L. L. Pewe, S. M. Kaech, J. T. Harty, Lung airway-surveilling CXCR3(hi) memory CD8(+) T cells are critical for protection against influenza A virus. Immunity 39, 939–948 (2013). 24238342 10.1016/j.immuni.2013.09.013 PMC3872058

[17] B. Slütter , Dynamics of influenza-induced lung-resident memory T cells underlie waning heterosubtypic immunity. Sci. Immunol. 2, eaag2031 (2017). 28783666 10.1126/sciimmunol.aag2031 PMC5590757

[18] N. Van Braeckel-Budimir, S. M. Varga, V. P. Badovinac, J. T. Harty, Repeated antigen exposure extends the durability of influenza-specific lung-resident memory CD8^+^ T cells and heterosubtypic immunity. Cell Rep. 24, 3374–3382.e3 (2018). 30257199 10.1016/j.celrep.2018.08.073 PMC6258017

[19] S. van de Wall, V. P. Badovinac, J. T. Harty, Influenza-specific lung-resident memory CD8^+^ T cells. Cold Spring Harb. Perspect. Biol. 13, a037978 (2021). 33288540 10.1101/cshperspect.a037978 PMC7849341

[20] M. A. Luangrath, M. E. Schmidt, S. M. Hartwig, S. M. Varga, Tissue-resident memory T cells in the lungs protect against acute respiratory syncytial virus infection. Immunohorizons 5, 59–69 (2021). 33536235 10.4049/immunohorizons.2000067 PMC8299542

[21] M. R. Olson, S. M. Hartwig, S. M. Varga, The number of respiratory syncytial virus (RSV)-specific memory CD8 T cells in the lung is critical for their ability to inhibit RSV vaccine-enhanced pulmonary eosinophilia. J. Immunol. 181, 7958–7968 (2008). 19017987 10.4049/jimmunol.181.11.7958 PMC2587004

[22] D. J. Gasper , Effective respiratory CD8 T-cell immunity to influenza virus induced by intranasal carbomer-lecithin-adjuvanted non-replicating vaccines. PLoS Pathog. 12, e1006064 (2016). 27997610 10.1371/journal.ppat.1006064 PMC5173246

[23] C. B. Marinaik , Programming multifaceted pulmonary T cell immunity by combination adjuvants. Cell Rep Med 1, 100095 (2020). 32984856 10.1016/j.xcrm.2020.100095 PMC7508055

[24] P. A. Szabo , Longitudinal profiling of respiratory and systemic immune responses reveals myeloid cell-driven lung inflammation in severe COVID-19. Immunity 54, 797–814.e6 (2021). 33765436 10.1016/j.immuni.2021.03.005 PMC7951561

[25] K. McMahan , Correlates of protection against SARS-CoV-2 in rhesus macaques. Nature 590, 630–634 (2021). 33276369 10.1038/s41586-020-03041-6 PMC7906955

[26] R. Channappanavar, C. Fett, J. Zhao, D. K. Meyerholz, S. Perlman, Virus-specific memory CD8 T cells provide substantial protection from lethal severe acute respiratory syndrome coronavirus infection. J. Virol. 88, 11034–11044 (2014). 25056892 10.1128/JVI.01505-14 PMC4178831

[27] J. Sun , Generation of a broadly useful model for COVID-19 pathogenesis, vaccination, and treatment. Cell 182, 734–743.e5 (2020). 32643603 10.1016/j.cell.2020.06.010 PMC7284240

[28] D. Planas , Sensitivity of infectious SARS-CoV-2 B.1.1.7 and B.1.351 variants to neutralizing antibodies. Nat. Med. 27, 917–924 (2021). 33772244 10.1038/s41591-021-01318-5

[29] Y. Weisblum , Escape from neutralizing antibodies by SARS-CoV-2 spike protein variants. eLife 9, e61312 (2020). 33112236 10.7554/eLife.61312 PMC7723407

[30] M. Hoffmann , SARS-CoV-2 variants B.1.351 and P.1 escape from neutralizing antibodies. Cell 184, 2384–2393.e12 (2021). 33794143 10.1016/j.cell.2021.03.036 PMC7980144

[31] L. G. Thorne , Evolution of enhanced innate immune evasion by SARS-CoV-2. Nature 602, 487–495 (2021). 34942634 10.1038/s41586-021-04352-y PMC8850198

[32] P. S. Arunachalam , Adjuvanting a subunit COVID-19 vaccine to induce protective immunity. Nature 594, 253–258 (2021). 33873199 10.1038/s41586-021-03530-2

[33] P. Jalkanen , COVID-19 mRNA vaccine induced antibody responses against three SARS-CoV-2 variants. Nat. Commun. 12, 3991 (2021). 34183681 10.1038/s41467-021-24285-4 PMC8239026

[34] X. Montagutelli , The B1.351 and P.1 variants extend SARS-CoV-2 host range to mice. *bioRxiv* [Preprint] (2021). https://www.biorxiv.org/content/10.1101/2021.03.18.436013v2 (Accessed 16 February 2022).

[35] E. Cameroni , Broadly neutralizing antibodies overcome SARS-CoV-2 omicron antigenic shift. *bioRxiv* [Preprint] (2021). https://www.biorxiv.org/content/10.1101/2021.12.12.472269v2 (Accessed 16 February 2022). 10.1038/s41586-021-04386-2 PMC9531318 35016195

[36] L. Liu , Striking antibody evasion manifested by the omicron variant of SARS-CoV-2. Nature 602, 676–681 (2021). 35016198 10.1038/s41586-021-04388-0

[37] J. M. Carreño , Activity of convalescent and vaccine serum against SARS-CoV-2 omicron. Nature 602, 682–688 (2021). 35016197 10.1038/s41586-022-04399-5

[38] G. Alter , Immunogenicity of Ad26.COV2.S vaccine against SARS-CoV-2 variants in humans. Nature 596, 268–272 (2021). 34107529 10.1038/s41586-021-03681-2 PMC8357629

[39] B. J. Laidlaw, J. E. Craft, S. M. Kaech, The multifaceted role of CD4(+) T cells in CD8(+) T cell memory. Nat. Rev. Immunol. 16, 102–111 (2016). 26781939 10.1038/nri.2015.10 PMC4860014

[40] W. Lee , Carbomer-based adjuvant elicits CD8 T-cell immunity by inducing a distinct metabolic state in cross-presenting dendritic cells. PLoS Pathog. 17, e1009168 (2021). 33444400 10.1371/journal.ppat.1009168 PMC7840022

[41] M. Gagne , Protection from SARS-CoV-2 Delta one year after mRNA-1273 vaccination in rhesus macaques coincides with anamnestic antibody response in the lung. Cell 185, 113–130.e15 (2021). 34921774 10.1016/j.cell.2021.12.002 PMC8639396

[42] M. M. Painter , Rapid induction of antigen-specific CD4^+^ T cells is associated with coordinated humoral and cellular immunity to SARS-CoV-2 mRNA vaccination. Immunity 54, 2133–2142.e3 (2021). 34453880 10.1016/j.immuni.2021.08.001 PMC8361141

[43] J. Mateus , Low-dose mRNA-1273 COVID-19 vaccine generates durable memory enhanced by cross-reactive T cells. Science 374, eabj9853 (2021). 34519540 10.1126/science.abj9853 PMC8542617

