# Supplementary material for: Vaccine-induced systemic and mucosal T cell immunity to SARS-CoV-2 viral variants
Source: Proc Natl Acad Sci U S A. 2022 May 13;119(20):e2118312119. doi: 10.1073/pnas.2118312119 (PMC9171754; doi:10.1073/pnas.2118312119)
Supplement: Supplementary File [file pnas.2118312119.sapp.pdf]

Supplemental Information

for

Vaccine-Induced Systemic and Mucosal T-Cell Immunity to SARS-CoV-2 Viral Variants

Brock Kingstad-Bakke, Woojong Lee, Shaswath S. Chandrasekar, David J. Gasper, Cristhian Salas-Quinchucua, Thomas Cleven, Jeremy A. Sullivan, Adel Talaat, Jorge E. Osorio and M. Suresh

Supplemental Figures 1-9

Supplemental Material & Methods

Supplemental Information Table 1

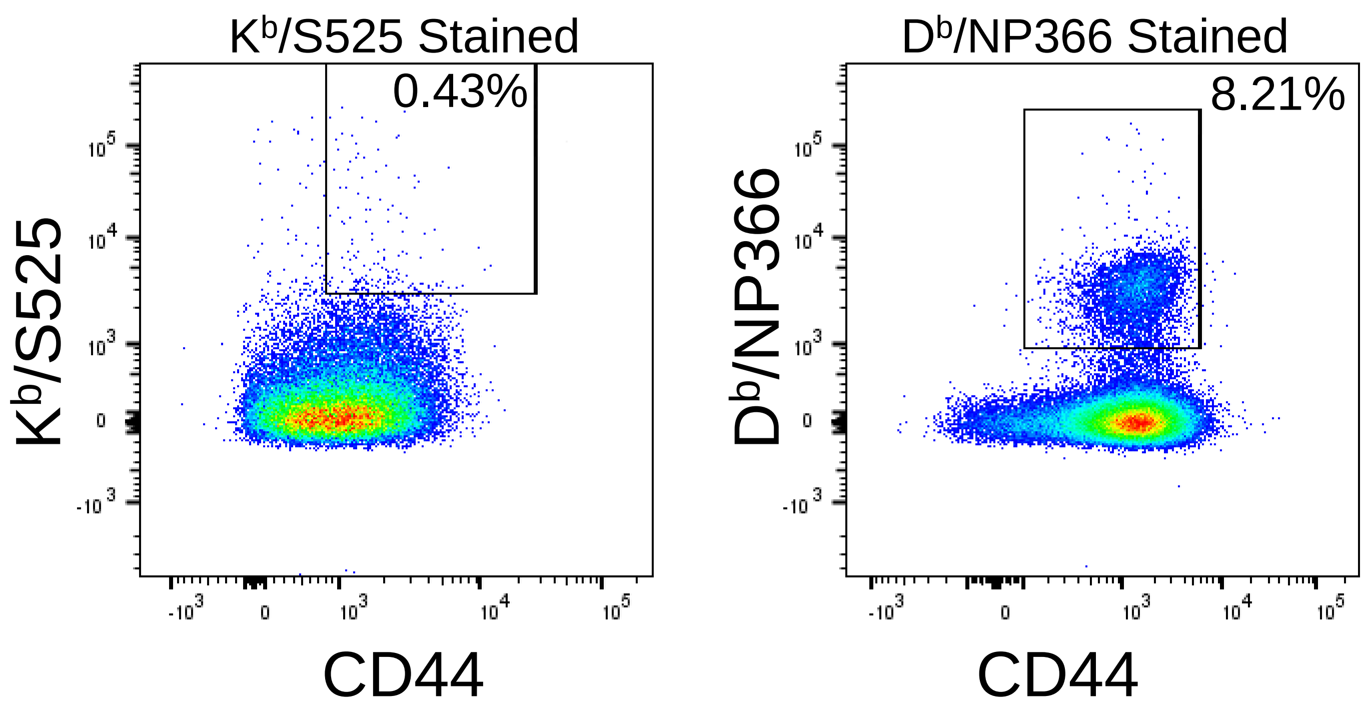

Supplemental Figure 1. Lack of K^b/S525 Tetramer Binding to Effector CD8 T Cells in Lungs of Influenza Virus-Infected Mice. C57BL/6 mice were infected intranasally with 50 PFU of the PR8/H1N1 influenza A virus. At day 10 after infection, single-cell suspensions from lungs were stained with viability dye, followed by D^b/NP366 (immunodominant epitope in the influenza virus nucleoprotein) or K^b/S525 (CTL epitope in SARS-CoV-2 spike protein) tetramers in combination with anti-CD8 and CD44 antibodies. FACS plots show percentages of S525 or NP366-tetramer-binding cells among CD8 T cells.

Supplemental Figure 2

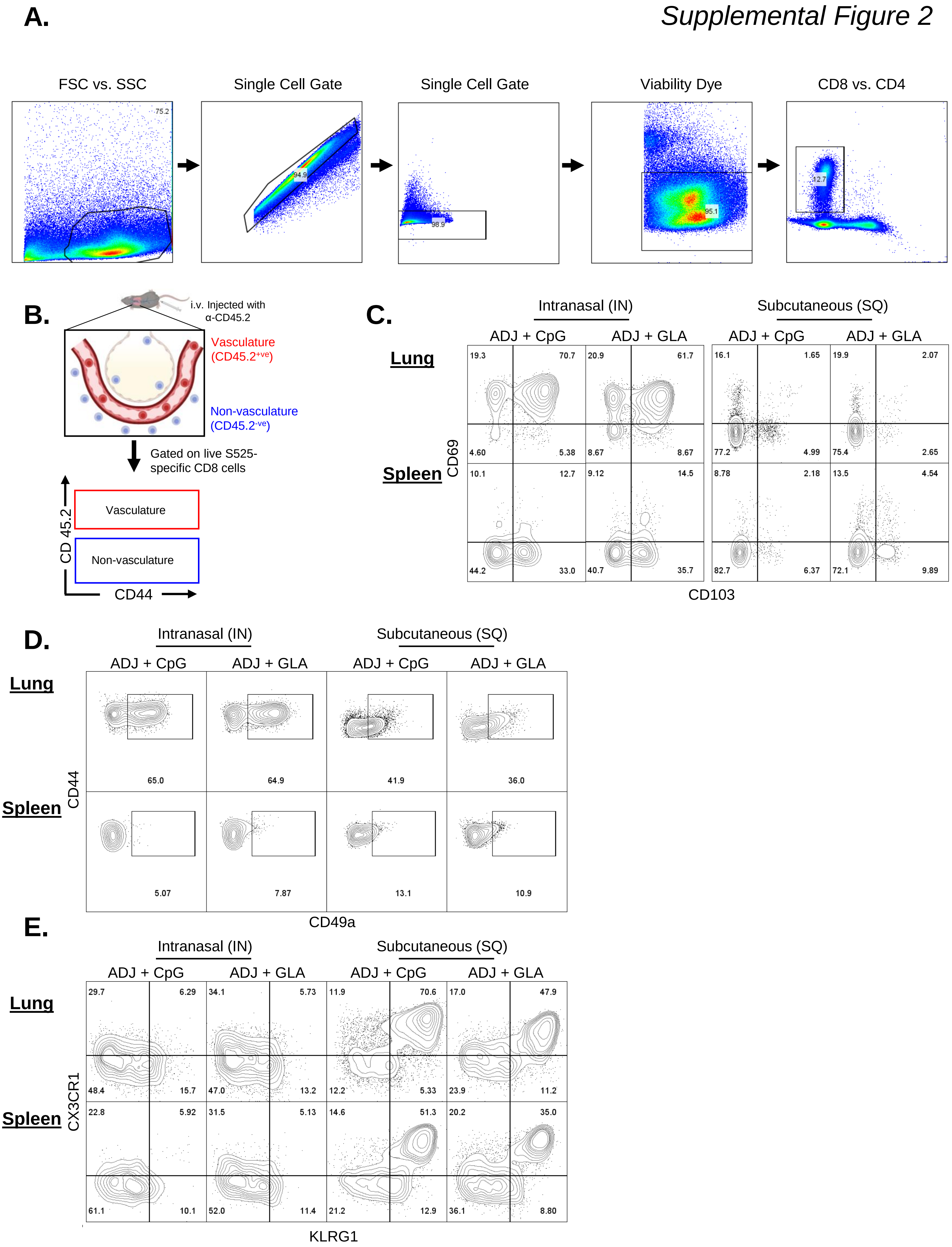

Supplemental Figure 2 cont.

F.

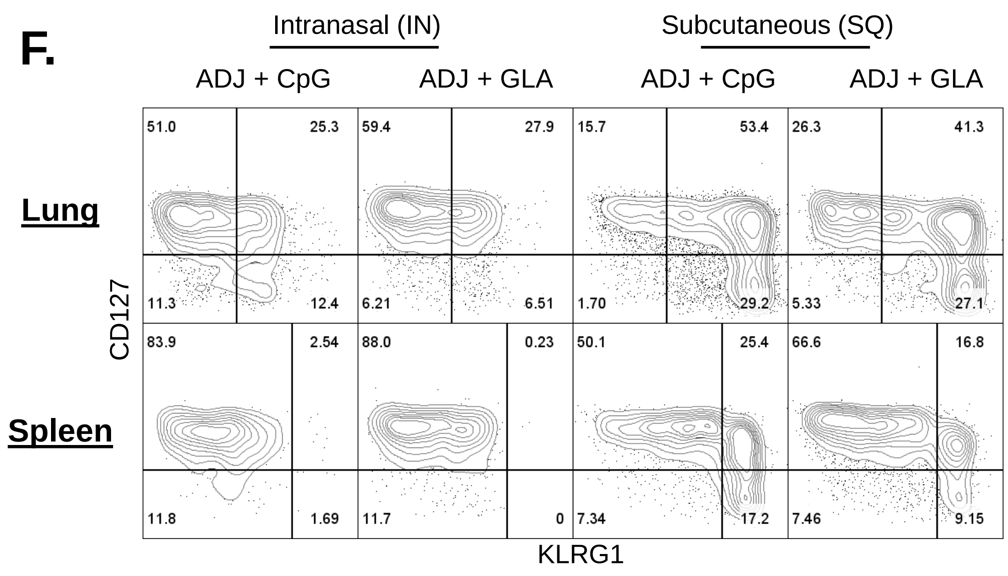

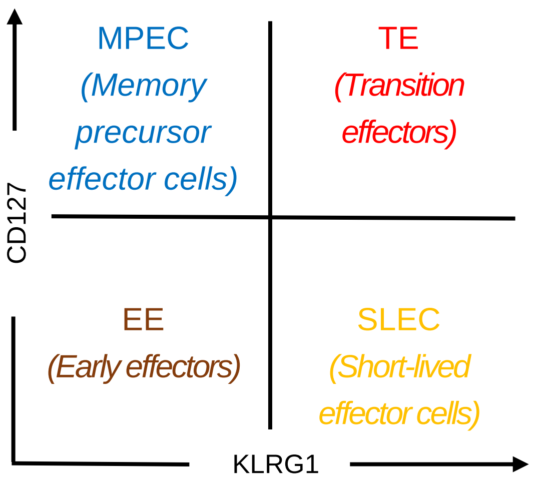

G.

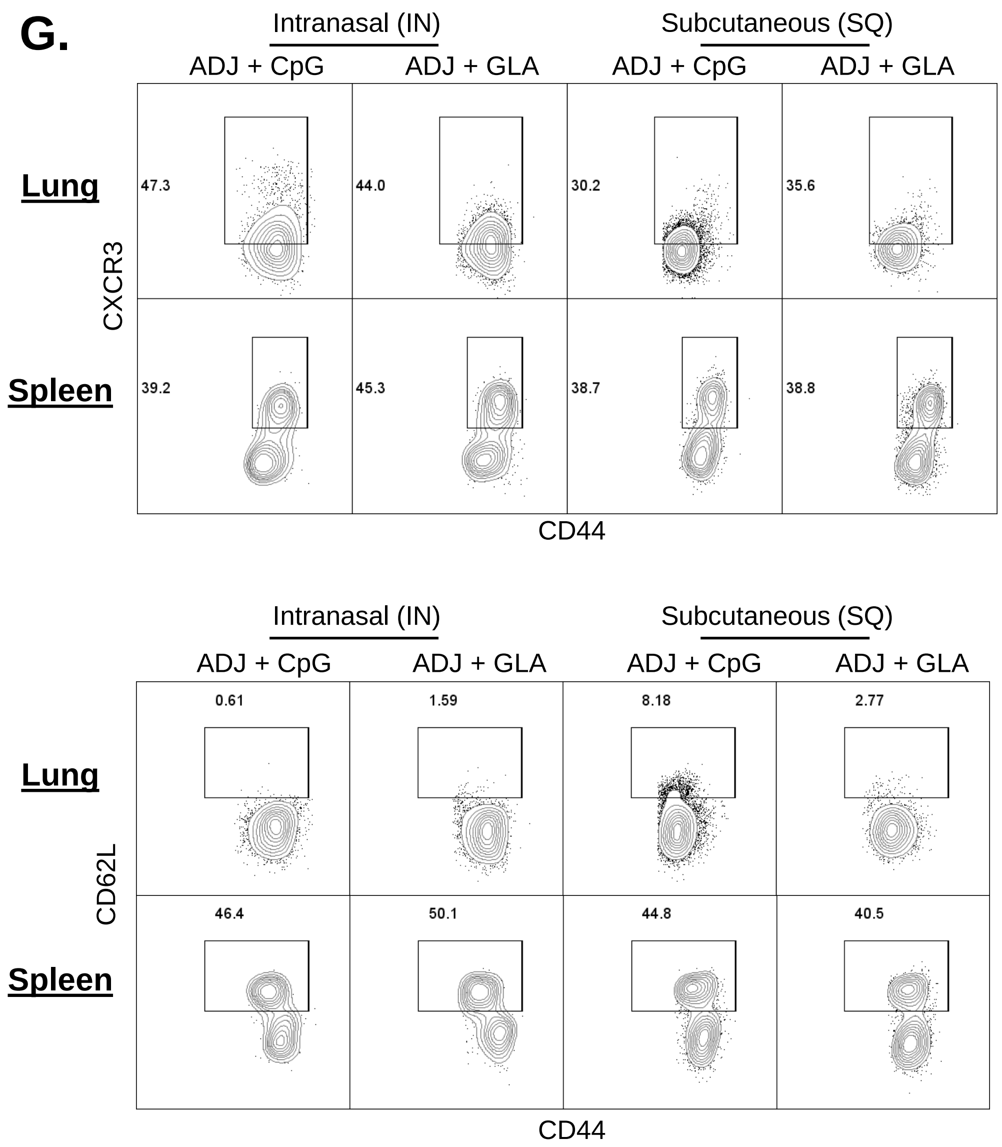

Supplemental Figure 2. Representative FACS Plots for Phenotypic Analysis of Effector K^b/S525-Specific CD8 T Cells from IN or SQ vaccinated mice. Mice were vaccinated twice with recombinant SARS-CoV-2 spike protein formulated in ADJ+CpG or ADJ+GLA by the IN or the SQ route. At day 8 after booster vaccination, phenotype of K^b/S525-specific CD8 T cells were analyzed by flow cytometry. (A) Gating strategy for visualization of K^b/S525-specific CD8 T cells: FSC. vs. SSC. for lymphocyte gate → singlets → live-cell gate → CD4 or CD8 T cells. (B-G) Lung cells and splenocytes were stained with K^b/S525 tetramer in conjunction with antibodies to the indicated cell surface molecules and analyzed by flow cytometry. Representative FACS plot for (C) CD69^{+ve}CD103^{+ve} (D) CD49a^{+ve}, (E) CX3CR1^{+ve}KLRG1^{+ve} (F) Short-lived effector cells (SLECs; CD127^{LO}/KLRG-1^{HI}), memory precursor effector cells (MPECs; CD127^{HI}/KLRG-1^{LO}) and transition effector cells (TEs; CD127^{Hi}/ KLRG1^{HI}), are represented and graphed. (G) CXCR3^{+ve} and CD62L$^{+ve\ cells}$ among S525-specific CD8 T cells in lung and spleen of vaccinated mice. Data are representative of 5 mice/group from two independent experiments.

Supplemental Figure 3

A.

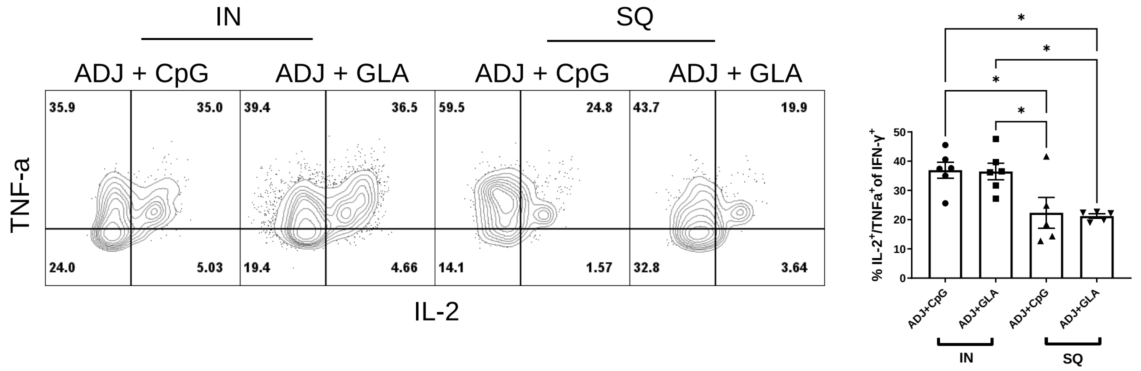

B.

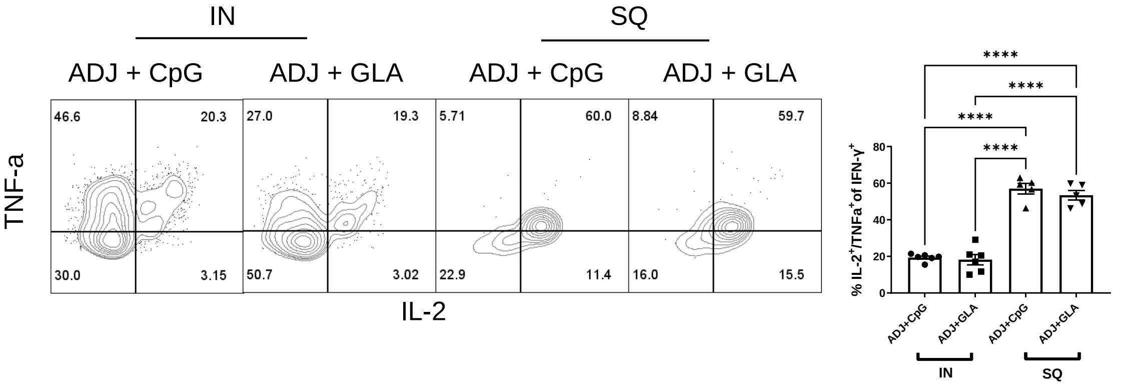

Supplemental Figure 3. Functional Polarization of Effector CD8 T Cells in Vaccinated Mice. Cohorts of C57BL/6 mice were vaccinated twice with recombinant SARS-CoV-2 spike protein formulated in ADJ+CpG or ADJ+GLA by the IN or the SQ route. At day 8 after booster vaccination, cytokine production by K^b/S525-specific CD8 T cells were analyzed by intracellular cytokine staining. Lung cells or splenocytes were stimulated with S525 peptide, as described in **Figure 3**. (A) Percentages of IL-2/TNF-α–producing cells among the gated IFN-γ–producing S525-specific CD8 T cells in lungs. (B) Percentages of IL-2/TNF-α–producing cells among the gated IFN-γ producing CD8 T cells in spleen. Data represent two independent experiments**.**

Supplemental Figure 4

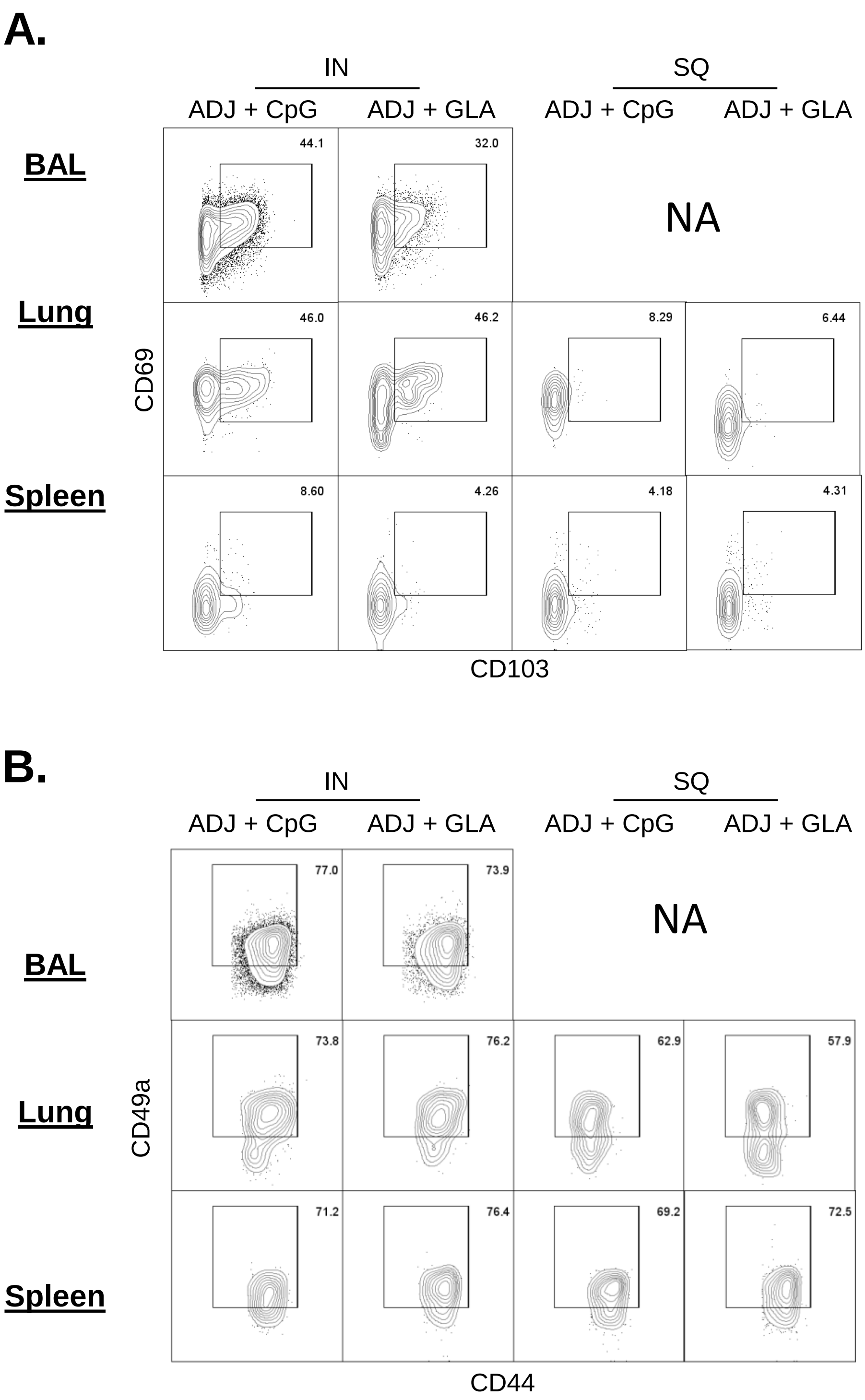

Supplemental Figure 4. Representative FACS Plots for Phenotypic and Functional Analysis of Memory S525-Specific CD8 T cells from IN or SQ Vaccinated Mice. Cohorts of C57BL/6 mice were vaccinated twice with recombinant SARS-CoV-2 spike protein formulated in ADJ+CpG or ADJ+GLA by the IN or the SQ route. At day 44 after booster vaccination, cell surface phenotype (A, B) of K^b/S525-specific CD8 T cells were analyzed by flow cytometry. (A-B) Representative FACS plots for (A) $CD69^{+ve}$ /$CD103^{+ve}$ (B) $CD49a^{+ve}$ among S525-specific CD8 T cells in lung and spleen of vaccinated mice. For SQ vaccinated mice, there were insufficient S525 specific CD8 T cells in airways to analyze as indicated by NA in the figure. Data are representative of 5 mice/group from two independent experiments.

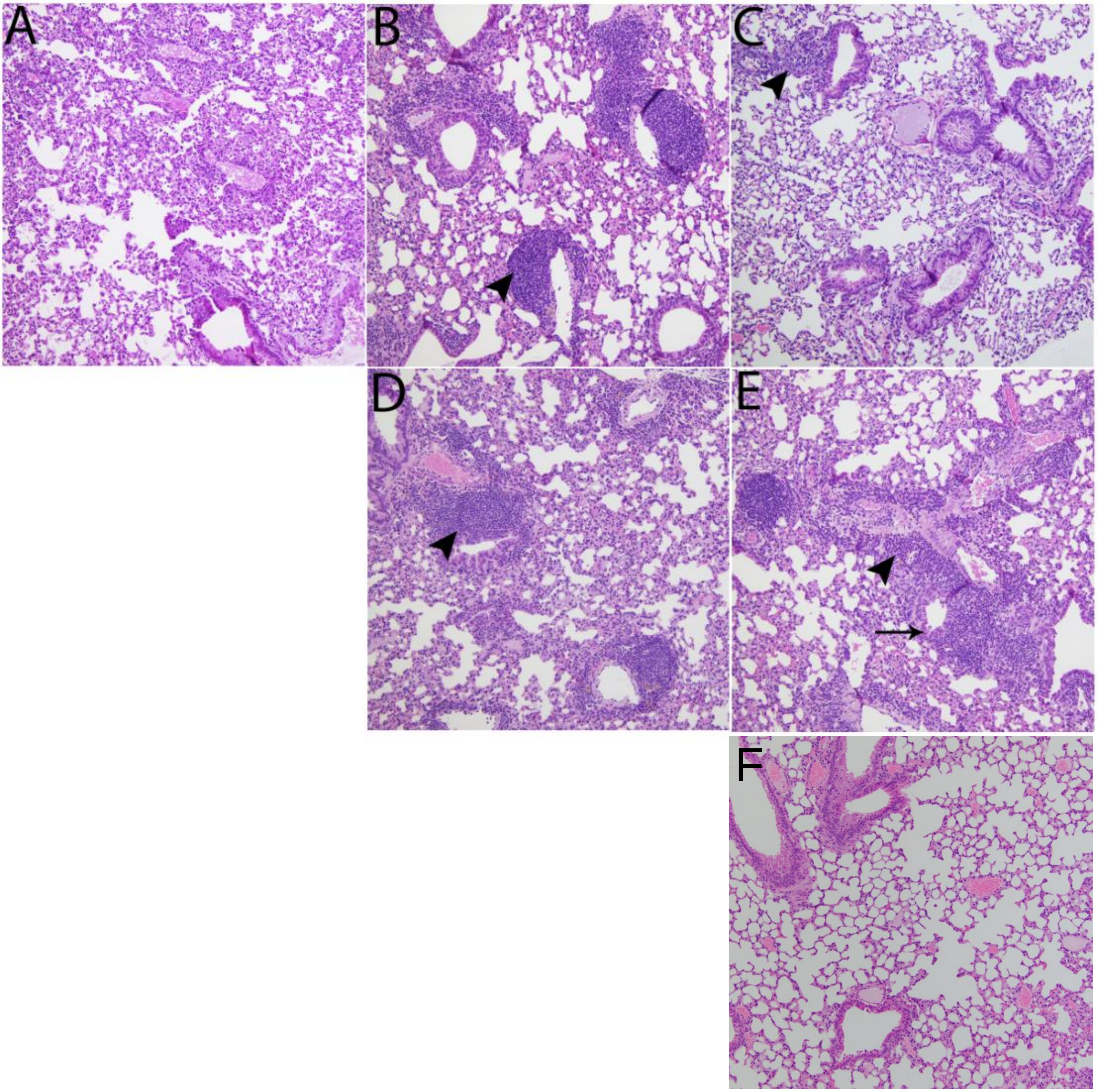
A
B
C
D
E
F

G.

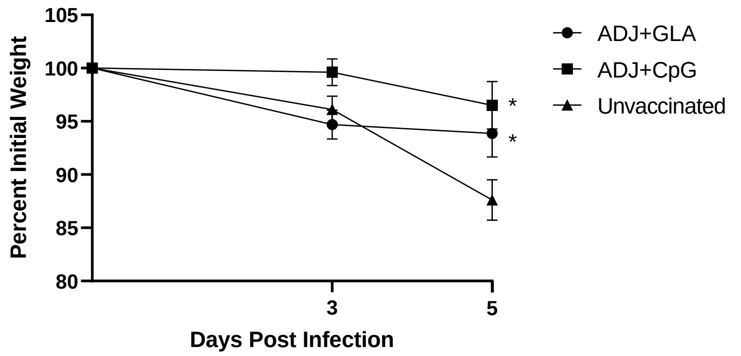
105
100
95
90
85
80
Percent Initial Weight
3
5
Days Post Infection
*
*
ADJ+GLA
ADJ+CpG
Unvaccinated

Supplemental Figure 5. Vaccines Protect Against SARS-CoV-2-Induced Lung Pathology and Weight loss. K18 ACE2 transgenic mice were vaccinated twice IN or SQ with SARS-Co-V-2 S protein formulated in ADJ+GLA or ADJ+CpG. At 50 days after booster vaccination, mice were challenged with the WA strain of SARS-CoV-2 virus; unvaccinated mice were challenged as controls. On day 5 after viral challenge, lung pathology was assessed by staining lung sections with Hematoxylin and Eosin. A. Unvaccinated. Moderate diffuse thickening of alveolar walls, mild increase in circulating interstitial lymphocytes, mild type II pneumocyte hyperplasia, moderate alveolar histiocytosis B. ADJ + CpG SQ. Mild alveolar wall thickening and mildly increased interstitial lymphocytes & macrophages. C. ADJ + GLA SQ. Alveolar walls are not thickened, but contain mildly increased numbers of lymphocytes, and nodular perivascular lymphoid aggregates are rare. D. ADJ + CpG IN. Moderate thickening of alveolar walls, moderate increase in interstitial lymphocytes and macrophages, and formation of nodular perivascular and peribronchiolar lymphoid aggregates. E. ADJ + GLA IN. Similar to D, with increased peribronchiolar lymphoid aggregates. B-E. Nodular perivascular (arrowhead) and peribronchiolar (arrow) lymphoid aggregates. HE stains, 100x magnification. F. Uninfected. Lungs from uninfected K18 ACE2 transgenic are included for comparison. G. Graph showing weight loss as a percent of initial starting weight over days following infection.

Supplemental Figure 6

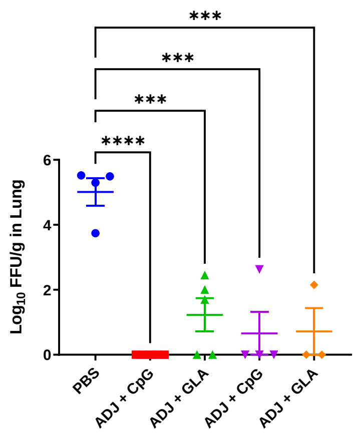

Supplemental Figure 6. Vaccine-Induced Protective Immunity to SARS-CoV2 Virus in Vaccinated and Adenovirus/ACE2-Sensitized C57BL/6 Mice. Cohorts of C57BL/6 mice were vaccinated twice with recombinant SARS-CoV-2 spike protein formulated in ADJ+CpG or ADJ+GLA by the IN or the SQ route. At 101 days after booster vaccination, mice were sensitized to SARS-CoV-2 infection by infecting with the hAce2-Adenovirus, then challenged 4 days later with the WA strain of SARS-CoV-2 virus; unvaccinated mice were challenged as controls. SARS-CoV-2 viral titers were quantified in the lungs on D5 after challenge.

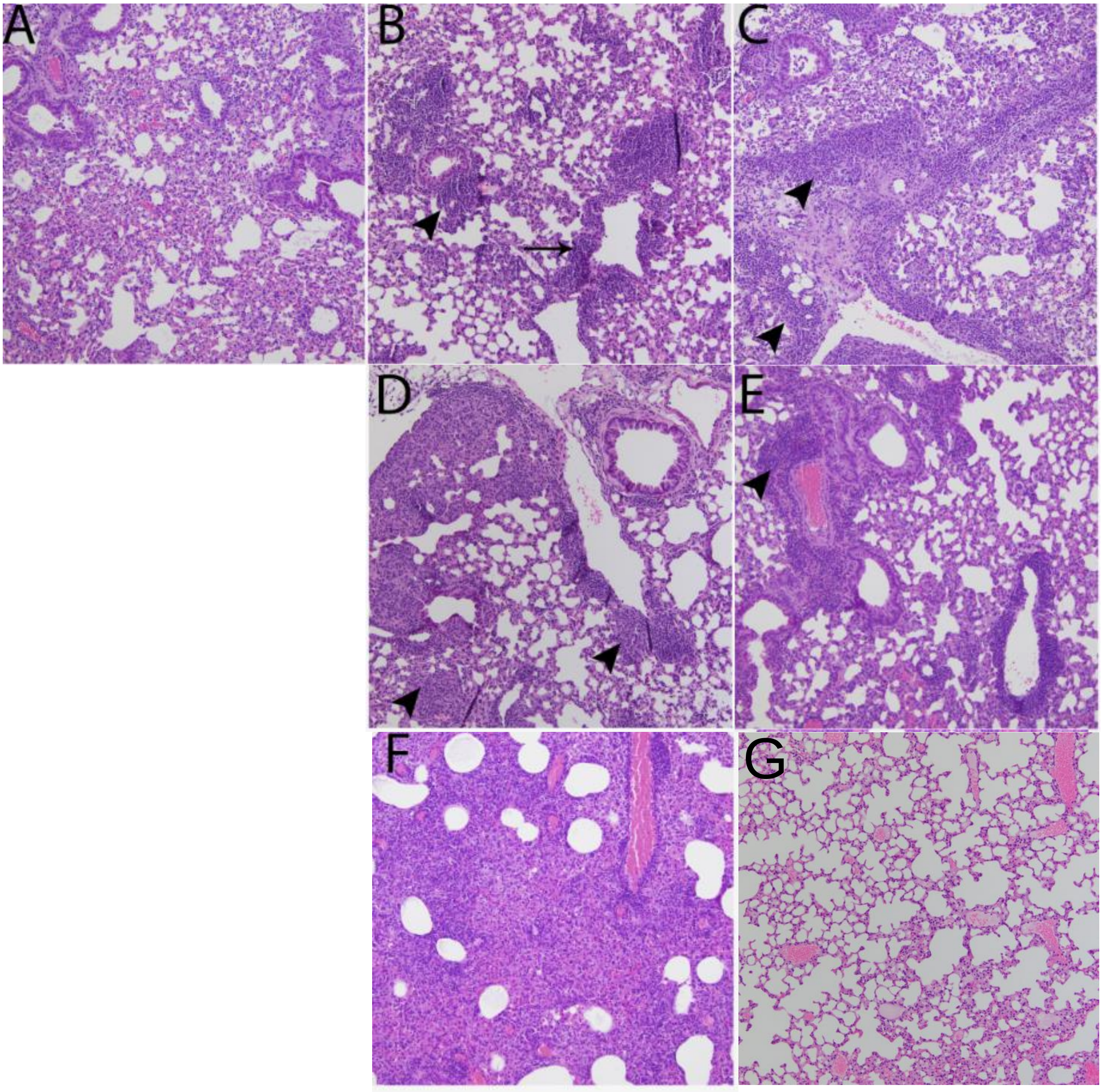
A
B
C
D
E
F
G

H.

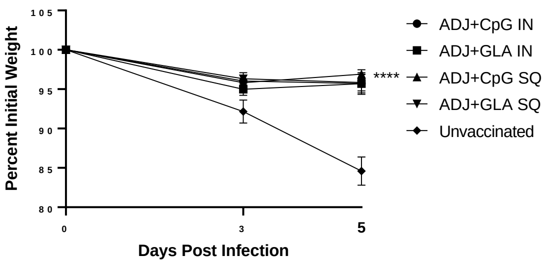
Percent Initial Weight
105
100
95
90
85
80
0
3
5
Days Post Infection
****
ADJ+CpG IN
ADJ+GLA IN
ADJ+CpG SQ
ADJ+GLA SQ
Unvaccinated

Supplemental Figure 7. Vaccine-Induced Protection Against Lung Pathology and Weight Loss Induced by the B.1.351 Variant of SARS-CoV-2. Cohorts of K18-hACE2 mice were vaccinated twice with S protein of the WA strain of SARS-CoV-2, as described in Figure 2. At 65 days after booster vaccination, mice were challenged with the B.1.351 variant of SARS-CoV-2 virus; unvaccinated mice were challenged as controls. A. Unvaccinated. Moderate to severe diffuse thickening of alveolar walls, mildly increased interstitial lymphocytes and macrophages, and moderate alveolar histiocytosis. B. ADJ + CpG SQ. Mild alveolar wall thickening and mildly increased interstitial lymphocytes & macrophages. C. ADJ + GLA SQ. Alveolar walls are not thickened, but contain mildly increased numbers of lymphocytes, and nodular perivascular lymphoid aggregates are rare. D. ADJ + CpG IN. Moderate thickening of alveolar walls, moderate increase in interstitial lymphocytes and macrophages, and formation of nodular perivascular and peribronchiolar lymphoid aggregates. E. ADJ + GLA IN. Representative of 7 of 11 lung samples. Moderate thickening of alveolar walls, moderate increase in interstitial lymphocytes and macrophages, and formation of nodular perivascular and peribronchiolar lymphoid aggregates. F. ADJ + GLA IN. Representative of multifocal lesions in 4 of 11 lungs. Severe organizing lobar pneumonia with loss of alveolar architecture. B-E. Nodular perivascular (arrowhead) and peribronchiolar (arrow) lymphoid aggregates. G. Lungs from uninfected K18 ACE2 transgenic are included for comparison. HE stains, 100x magnification. H. Graph showing weight loss as a percent of initial starting weight over days following infection.

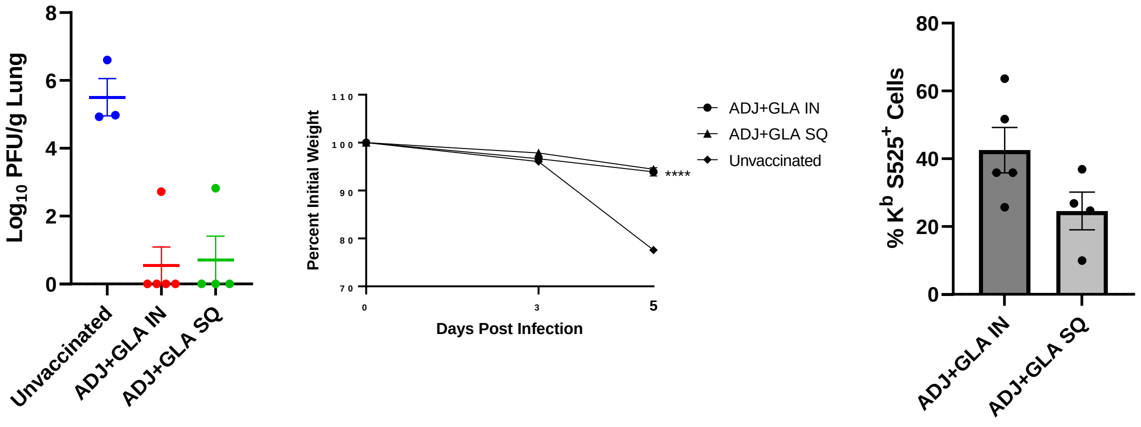

Supplemental Figure 8. Durable Protective Immunity to the South African Variant of SARS-CoV-2. Six-eight-week-old K18-hACE2 mice were vaccinated twice IN or SQ with SARS-Co-V-2 S protein formulated in ADJ+GLA or ADJ+CpG. At 140 days after booster vaccination, when mice were ~27 weeks of age, they were challenged with the SA strain of SARS-CoV-2 virus; unvaccinated mice were challenged as controls. On day 5 after viral challenge, viral titers and S525-specific CD8 T cells were quantified in the lungs. Graphs show viral titers in lungs, weight loss, and recall S525-specific CD8 T-cell responses in lungs.

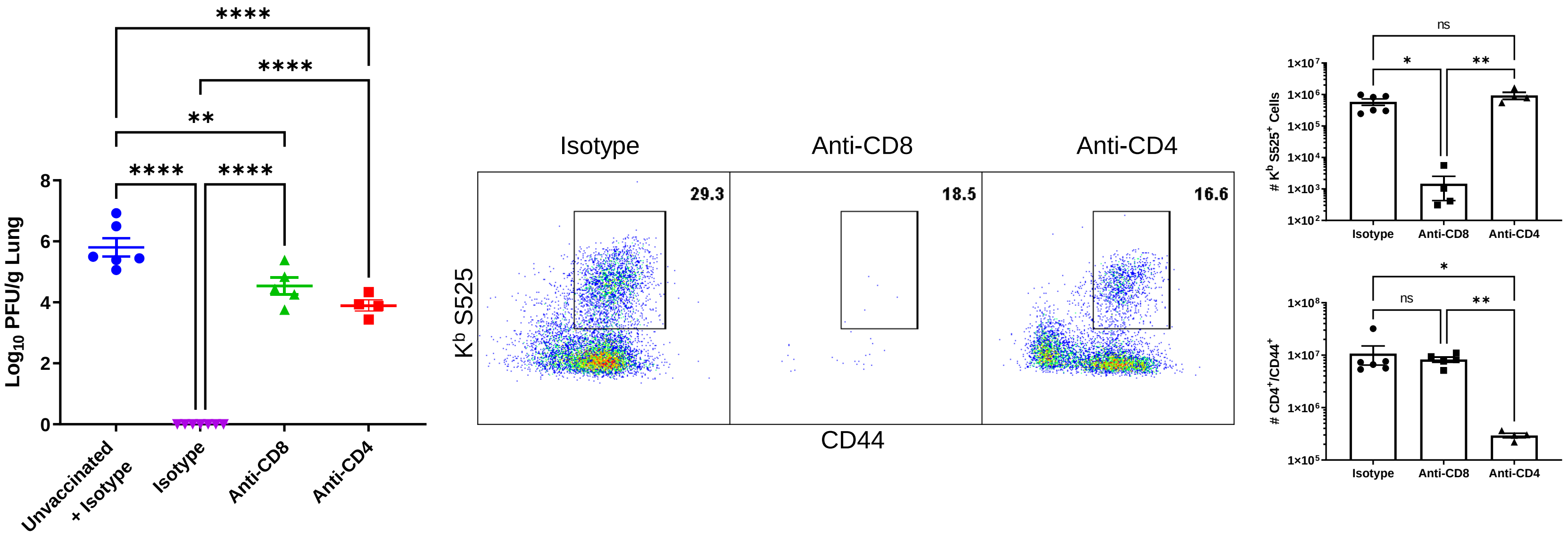

Supplemental Figure 9. T-Cell dependent Vaccine-Induced Protective immunity to the South African variant of SARS-CoV-2 in C57BL/6 mice. Cohorts of 6-8-week-old C57BL/6 mice were vaccinated twice with S protein of the WA strain of SARS-CoV-2, as described in Figure 2. At 154 days after booster vaccination, when mice were ~31 weeks of age, vaccinated mice were treated with isotype control antibodies, anti-CD4 antibodies or anti-CD8 antibodies (prior to and during viral challenge) and challenged with the B.1.351 variant of SARS-CoV-2 virus; unvaccinated mice were challenged as controls. Viral titers and S525-specific CD8 T cells were quantified in the lungs on day 5 after challenge. FACS plots are gated on CD8 T cells and graphs show total numbers of S525-specific CD8 T cells.

Supplemental Methods

Tissue processing and Flow cytometry

Spleens and lungs were processed into single cell suspensions using mechanical digestion and standard collagenase-based methods, as previously described (1). To stain for surface markers, single-cell suspensions were first stained for viability with Ghost Dye™ Red 780 (Tonbo Biosciences, stained with antibodies and tetramers diluted in Brilliant Stain Buffer (BSB, BD Biosciences) for 60 minutes at 4C, and fixed with 2% paraformaldehyde. All samples were acquired on LSRFortessa (BD Biosciences) and analyzed with FlowJo V.10 software (TreeStar, Ashland, OR).

Intracellular staining for transcription factors

To stain for intracellular factors directly *ex vivo*, single-cell suspensions were stained for viability as above. Next, samples were stained with antibodies and tetramers diluted in BSB (BD Biosciences), which were then fixed, permeabilized and subsequently stained for using the transcription factors staining kit (eBioscience) with the antibodies (indicated in **Supplemental Table 1)** diluted in eBioscience Perm Wash buffer. All samples were acquired on LSRFortessa (BD Biosciences) and analyzed with FlowJo V.10 software (TreeStar, Ashland, OR).

Intracellular Cytokine Staining (ICCS)

For intracellular cytokine staining, cells were plated and stimulated for 5 hours at 37C in the presence of brefeldin A (1 µl/ml, GolgiPlug, BD Biosciences), human recombinant IL-2 (10 U/well) and with or without S525 (Genscript) at 1ug/ml, or Peptide Array SARS-CoV-2 Spike (S) Glycoprotein (BEI, NR-52402). After stimulation, cells were transferred to 96-well round bottom plate (Corning) and stained for viability dye as above, and fixed/permeabilizated with Cytofix/Cytoperm kit (BD Biosciences, Franklin Lakes, NJ) according to manufacturer's protocol.

Samples were stained with antibodies corresponding to cytokines indicated in **Supplemental Table 1** in perm wash buffer. All the staining procedures were performed on ice.

Ex vivo stimulation for cytokine analysis

For intracellular cytokine staining, cells were plated and stimulated for 5 hours at 37C in the presence of brefeldin A (1 µl/ml, GolgiPlug, BD Biosciences), human recombinant IL-2 (10 U/well) and with or without S525 (Genscript) at 1ug/ml, or Peptide Array SARS-CoV-2 Spike (S) Glycoprotein (BEI, NR-52402). After stimulation, cells were stained as described previously(1) with antibodies (indicated in **Supplemental Table 1)**.

Cells and Viruses

African Green Monkey Kidney Cells (Vero), and Human Embryonic Kidney 293T (HEK293T) cells were obtained from ATCC (ATCC; Manassas, VA, USA). SARS-CoV-2 USA-WA1/2020 (WA strain) and hCoV-19/South Africa/KRISP-EC-K005321/2020 (SA strain) viruses used in these studies were obtained from BEI resources (NR-52281, NR-55282 respectively), and propagated in Vero cells. Adenovirus Serotype 5, co-expressing recombinant human ACE2 (Ad5-hAce2) was obtained from BEI resources (NR-52390) and propagated in HEK293T cells and purified as previously described (2). SARS-CoV-2 WA strain viral titrations were performed by a focus forming unit assay with some modifications (3). SARS-CoV-2 SA strain viral titrations were performed similarly, but cells were incubated with virus for 5 days, fixed, and plaque forming units were calculated from observing cytopathic effects under a light microscope. SARS-CoV-2, isolate USA-WA1/2020 (lineage A), or isolate SA/2020 (lineage B.1.351) was propagated and titrated on Vero E6 cells. Serial dilutions were made from serum (1:20-1:14,580; 3-fold) and BAL (Undiluted-1:80; 2-fold) in serum-free Opti-MEM media and incubated with 100 PFU per well of SARS-CoV-2 isolates for 60 mins at 37°C and transferred into wells pre-seeded with Vero E6 cells. Plates were incubated at 37°C for 3-4 days before scoring for the cytopathic effect. Neutralization titer was calculated as the reciprocal of the highest dilution at which virus neutralization occurred.

Viral challenge

For all challenge studies, mice were euthanized on day 5 post challenge and lungs collected for viral titers (left lobe), histology, (a section of the inferior lobe) and T-cell analysis (remainder of lung tissue). For virus titration, lungs were weighed, then homogenized in Opti-MEM media containing 3% FBS via bead beating, clarified by centrifugation and titrated as described above. Lung sections for histology were taken from uninflated lungs and fixed in 10% neutral buffered formalin, sectioned, and stained with Hematoxylin and Eosin (H&E) by conventional methods.

1. C. B. Marinaik *et al.*, Programming Multifaceted Pulmonary T Cell Immunity by Combination Adjuvants. *Cell Rep Med* **1**, 100095 (2020).
2. M. A. Croyle, D. J. Anderson, B. J. Roessler, G. L. Amidon, Development of a highly efficient purification process for recombinant adenoviral vectors for oral gene delivery. *Pharmaceutical development and technology* **3**, 365-372 (1998).
3. Y. Ambuel *et al.*, A rapid immunization strategy with a live-attenuated tetravalent dengue vaccine elicits protective neutralizing antibody responses in non-human primates. *Front Immunol* **5**, 263 (2014).

Supplemental Table 1. List of antibodies used in the manuscript

Antibodies used at 1:200 concentration unless otherwise indicated

Antibody (Dilution Factor)	Company	Catalogue number
Rat anti-mouse CD8α-BUV395-	BD Biosciences	563786
Rat anti-mouse CD4-BUV496-	BD Biosciences	564667
Rat anti-mouse CD44-BV510-	BD Biosciences	563114
Rat anti-mouse CD62L-PE-CF594-	BD Biosciences	562404
Hamster anti-mouse-CD69-PE-Cy7-	BD Biosciences	553237
Hamster Anti-KLRG1-BV711-	BD Biosciences	564014
Rat anti-mouse IFN-γ-APC- (1:400)	BD Biosciences	554413
Rat anti-mouse TNF-α-BV421- (1:400 or 1:500)	BD Biosciences	554413
Rat anti-CD127-BV605-	Biolegend	135041
Mouse anti-CX3CR1-BV785-	Biolegend	SA011F11
Rat anti-IL-17A-FITC	Biolegend	506908
Rat anti-IL-2 PE/Dazzle 594-	Biolegend	503840
Hamster Anti-CD49a-BV605-	BD Pharmingen	740375
Hamster Anti-KLRG1-BV711-	BD Pharmingen	564014
Rat Anti-CD62L-Alexa 700-	BD Pharmingen	560517
Rat Anti-IL-2-PE-CF594-	BD Pharmingen	562483
Hamster Anti-CD279 (PD-1)-BV 650-	BD Pharmingen	744546
Hamster Anti-Mouse CD69-PE-Cy7	BD Pharmingen	552879
Rat Anti- CD4-APC-	BD Pharmingen	553051
Hamster Anti- CD103 Antibody-FITC	eBioscience	11-1031-85
Mouse anti-CD45.2 Monoclonal Antibody	eBioscience	12-0454-82

Mouse anti-Granzyme B-PE 1:10	Thermo Fisher Scientific	MHGB04
Rat anti-CD127-BV650- 1:150	Biolegend	135043
Mouse anti-CX3CR1-BV785	Biolegend	SA011F11
APC H2-D^b tetramers bearing the NP peptide NP366 1:150	NIH Tetramer Core Facility at Emory University	N/A
APC H2-K^b tetramers bearing the Spike peptide S525 1:150	NIH Tetramer Core Facility at Emory University	N/A
